# Genome-Wide Identification of *Pseudomonas aeruginosa* Genes Important for Desiccation Tolerance on Inanimate Surfaces

**DOI:** 10.1128/msystems.00114-22

**Published:** 2022-04-26

**Authors:** Sardar Karash, Timothy L. Yahr

**Affiliations:** a Department of Microbiology and Immunology, University of Iowa, Iowa City, Iowa, USA; University of Wisconsin-Madison

**Keywords:** *Pseudomonas aeruginosa*, desiccation, Tn-seq, stress responses, cell envelope, pyrimidine, reservoir

## Abstract

Pseudomonas aeruginosa is an opportunistic pathogen prevalent in the environment and in health care settings. Transmission in the health care setting occurs through human-human interactions and/or contact with contaminated surfaces. Moist surfaces such as respirators, sink and tub drains, and even disinfectants can serve as reservoirs. Dry surfaces such as plastic and stainless steel could also serve as a reservoir but would necessitate some degree of tolerance to desiccation. Using an assay to measure P. aeruginosa tolerance to desiccation on plastic and stainless-steel surfaces, we found that only 0.05 to 0.1% of the desiccated cells could be recovered 24 h postdesiccation. We took advantage of the strong selection imposed by desiccation to identify genes important for tolerance using Tn-seq. A highly saturated Tn-seq library was desiccated on plastic and stainless-steel surfaces. NexGen sequencing of the recovered cells identified 97 genes important for survival. Comparing cells desiccated under low- and high-nutrient conditions allowed for differentiation of genes important for desiccation tolerance. The 53 genes identified in the latter analysis are involved in maintenance of cell envelope integrity, purine and pyrimidine biosynthesis, tricarboxylic acid (TCA) cycle, and the hydrolysis of misfolded proteins. The Tn-seq findings were validated by competition experiments with wild-type (WT) cells and select Tn insertion mutants. Mutants lacking *carB* and *surA* demonstrated the largest fitness defects, indicating that pyrimidine biosynthesis and outer membrane integrity are essential for desiccation tolerance. Increased understanding of desiccation tolerance could provide insight into approaches to control environmental reservoirs of P. aeruginosa.

**IMPORTANCE** Health care-associated infections (HAIs) caused by Pseudomonas aeruginosa result in significant morbidity and mortality and are a significant economic burden. Moist environments that promote biofilm formation are an important reservoir for P. aeruginosa. Dry environments may also serve as a reservoir but would require some degree of desiccation tolerance. Here, we took a genome-wide approach to identify genes important for desiccation tolerance on plastic and stainless-steel surfaces. Genes involved in assembly of outer membrane proteins and pyrimidine biosynthesis were particularly important. Strains lacking these functions were unable to tolerate surface desiccation. These findings suggest that inhibitors of these pathways could be used to prevent P. aeruginosa survival on dry surfaces.

## INTRODUCTION

Pseudomonas aeruginosa is a Gram-negative opportunistic pathogen and a leading cause of nosocomial and health care-associated infections (HAIs). Common HAIs include pneumonia, urinary tract, surgical site, and bloodstream infections (1). Factors that contribute to the prevalence of these infections are the immunosuppressed status of the patients, the intrinsic resistance of P. aeruginosa to antimicrobials and disinfectants, and the prevalence of P. aeruginosa in the environment (2). Reservoirs for P. aeruginosa in the health care setting include moist surfaces such as sinks, faucets, drains, and medical equipment such as ventilators and endoscopes (3–6). Contamination of these surfaces is promoted by the propensity of P. aeruginosa to grow as biofilms, which have enhanced resistance properties that hamper decontamination efforts. An important component of infection control in the health care setting is reducing patient exposure to P. aeruginosa through hand hygiene measures, regular cleaning of patient rooms and shared equipment, and water quality management plans (7).

Another potential reservoir for P. aeruginosa is dry surfaces such as cloth, plastic, stainless steel, and flooring materials (8–13). Persistence on dry surfaces requires tolerance to desiccation. The responses to desiccation are complex and vary in efficiency between different bacterial species. Water loss triggers a cascade of events that pose a challenge to survival, including cell shrinkage, reduced membrane fluidity, macromolecular crowding, increased salt levels, and oxidative and metabolic stress (14, 15). Inherent features of an organism can promote tolerance. For instance, differences in the cell wall architecture contribute to the significant increase in the resistance of Gram-positive bacteria to drying relative to that of Gram-negative organisms (16, 17). Challenges associated with water loss can also be dealt with through adaptive responses. A common response is replacement of water with compatible solutes, such as glycerol or trehalose, that are either taken up from the environment or synthesized *de novo*. Compatible solutes stabilize membranes and proteins and counterbalance the osmolarity to protect cells from osmotic stress. Other induced responses include production of proteins that scavenge reactive oxygen species, DNA repair systems, phospholipid modifications that preserve membrane fluidity, biofilm formation, and extracellular polymeric substances (EPS) (14, 15). EPS production is one of the few factors known to contribute to P. aeruginosa desiccation tolerance. P. aeruginosa is a common pathogen isolated from the sputum of cystic fibrosis (CF) patients (18). CF isolates usually demonstrate a mucoid phenotype resulting from the overproduction of the polysaccharide alginate. Alginate is a secreted polymer of guluronic acid and mannuronic acid and is thought to facilitate localized retention of water to promote desiccation tolerance (19). Aside from alginate, our understanding of P. aeruginosa genes that contribute to desiccation tolerance on dry surfaces is limited.

Transposon mutagenesis coupled with next-generation sequencing (Tn-seq) is a powerful approach used to identify factors required for a phenotype of interest on a genome-wide scale. Tn-seq has been used to identify P. aeruginosa genes critical for fitness during biofilm growth, antibiotic tolerance, and growth survival during energy-limited growth arrest (20–22). In this study, we found that desiccation of log-phase-grown P. aeruginosa on either plastic or stainless-steel surfaces effectively kills all of the bacteria within 24 h. Desiccation of cells grown to stationary phase, however, is permissive for the survival of some cells up to 7 days following the time of desiccation. We took advantage of the selective pressure imposed by desiccation by performing a Tn-seq screen for genes important for desiccation tolerance. We identified 53 critical genes involved in desiccation tolerance. Competition experiments validated many of the genes identified by the Tn-seq approach. Two genes in particular, *surA* and *carB*, were essential for desiccation tolerance and function in outer membrane assembly and nucleotide biosynthesis, respectively. Cataloging genes required for desiccation tolerance is the first step toward understanding the response of P. aeruginosa to desiccation and devising strategies to exploit that sensitivity.

## RESULTS

### Growth to stationary phase is required for tolerance to desiccation on plastic and stainless-steel surfaces.

**FIG 1:**
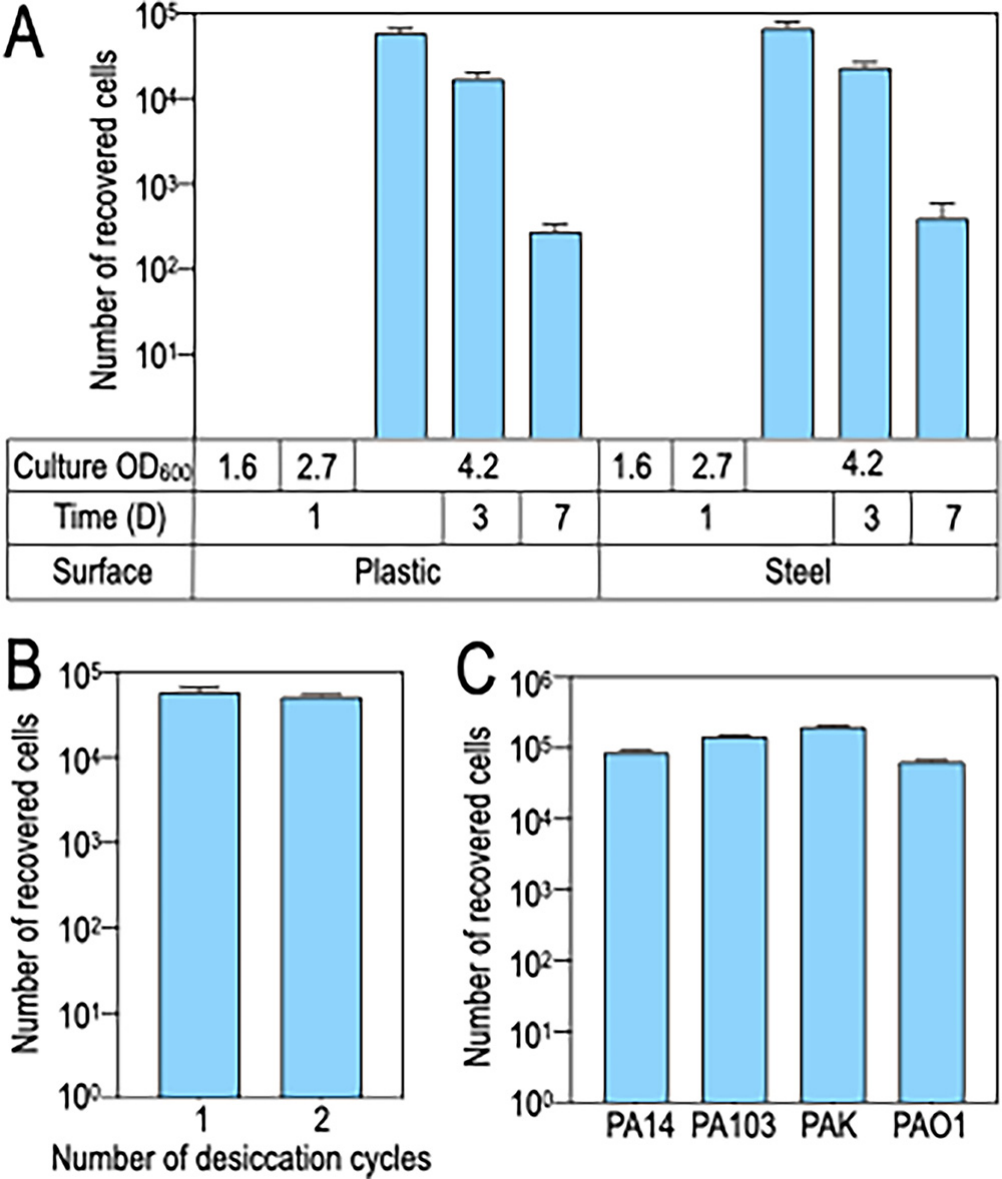
Survival of P. aeruginosa following desiccation on plastic and stainless-steel surfaces. (A) P. aeruginosa was cultured in LB to mid-exponential phase (OD_600_ 1.6), late-exponential phase (OD_600_ of 2.7), or stationary phase (OD_600_ of 4.2). Cells (10^8^ cells) were then harvested, washed with PBS, thinly spread onto the surface of either plastic or steel, allowed to air dry, and incubated at room temperature for 1, 3, or 7 days (D). At the indicated days, the cells were recovered by hydrating the surface with PBS and physically dislodging with a cell scraper and then enumerated by serial dilution and plating. (B) P. aeruginosa was desiccated for 1 day on plastic and recovered as described above. The recovered cells were cultured to stationary phase and then desiccated a second time. There was no significant difference between the numbers of cells recovered from the first and second desiccation assays. (C) P. aeruginosa strains PA14, PA103, PAK, or PAO1 were desiccated for 1 day on plastic and recovered as described above. Each experiment represents the average of at least three replicates.

We initially examined the tolerance of P. aeruginosa strain PA14 to desiccation on a plastic surface. Stationary-phase-grown cells were diluted 1:2,500 in fresh LB medium and cultured to early-exponential phase (optical density at 600 nm [OD_600_] of 0.6). Cells (10^8^ CFU/1 mL phosphate-buffered saline [PBS]) were collected by centrifugation, resuspended in PBS, spread onto the surface of plastic petri dishes, desiccated in a biological hood for 3 h, and then incubated at room temperature for 24 h. Cells were recovered by rehydrating the surface with PBS and scraping and enumerated by serial dilution on LB agar plates. Surprisingly, no viable cells were recovered (data not shown). We next tested cells grown to mid-exponential phase (OD_600_ of 1.6), late-exponential phase (OD_600_ of 2.7), or stationary phase (OD_600_ of 4.2) using the same conditions described above. Only cultures grown to stationary phase prior to desiccation yielded viable cells following the 24-h incubation period (Fig. 1A). Approximately 10^4^ to 10^5^ of the 10^8^ desiccated cells were recovered. To examine this further, the desiccation period was extended to 3 and 7 days. While a 3-day desiccation period had only a modest impact on the recovery of viable cells, extending that period to 7 days resulted in an ~100-fold reduction in cell viability. The findings were similar when cells were desiccated on stainless steel with survival being dependent upon the use of stationary-phase cells and viability decreasing when the desiccation period was extended to 7 days (Fig. 1A). To determine whether cells that survived desiccation had mutations that confer a fitness advantage, viable cells recovered from the first desiccation assay on plastic were desiccated a second time on plastic using the same experimental approach. The number of cells recovered was similar to that of the initial experiment, suggesting that desiccation survival does not confer a heritable fitness advantage (Fig. 1B). The recovery of only 0.05 to 0.1% of the desiccated PA14 cells from plastic or steel made us question whether other laboratory strains of P. aeruginosa have a similar level of desiccation tolerance. Desiccation of strains PA14, PA103, PAK, and PAO1 on plastic followed by recovery the next day resulted in recovery rates ranging from 0.04 to 0.1% (Fig. 1C).

### Identification of genes required for desiccation tolerance using Tn-seq.

The strong selection for desiccation tolerance was combined with Tn-seq to identify genes important for desiccation survival on plastic and stainless-steel surfaces. A highly saturated transposon mutant library was generated in P. aeruginosa strain PA14 using a Mariner-based transposon that preferentially targets TA dinucleotides (23). Preparation of libraries for next-generation sequencing was performed by amplification of transposon-genomic junctions using linear PCR, followed by c-tailing, and addition of barcodes and adaptors as reported previously (24). As described below, two Tn-seq experiments were performed in this study. The control samples from the second experiment were sequenced to a greater depth and used to assess the quality of the Tn-seq library. Of the 100,931 TA sites in the PA14 genome, 88,425 TA sites (87.6%) had insertions in our library (Table S1B). No obvious transposition hot spots were observed in the genome, suggesting that the transposon inserts randomly and in an unbiased manner at TA sites. A Hidden Markov Model (25) suggested that 5,382 (5.3%) of the TA sites correspond to essential genes (328 genes), which is consistent with previous estimates of the number of essential P. aeruginosa genes (26). Our transposon mutant library, therefore, contains insertions in 92.5% of the TA sites located in nonessential regions, 82% of which correspond to open reading frames (ORFs), while the remaining 18% are located in intergenic regions. Insertions at non-TA sites occurred at a low rate (3.5%) and were excluded from the analyses.

10.1128/msystems.00114-22.8 TABLE S1 Number of reads and insertions used in Tn-seq experiments. Download Table S1, TIF file, 2.5 MB. Copyright © 2022 Karash and Yahr. 2022 Karash and Yahr. https://creativecommons.org/licenses/by/4.0/ This content is distributed under the terms of the Creative Commons Attribution 4.0 International license.

**FIG 2:**
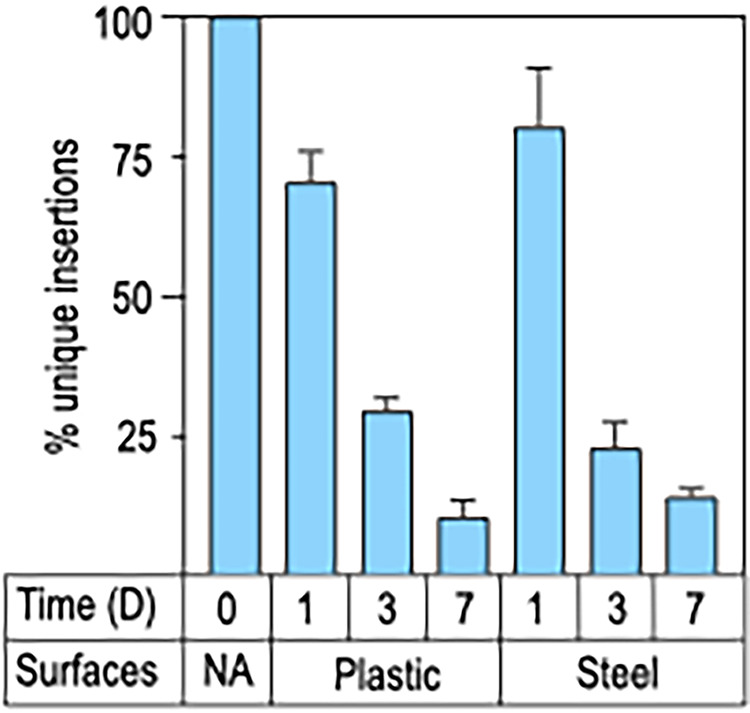
Genes required for desiccation tolerance on plastic and stainless-steel surfaces. The transposon mutant library grown to stationary phase was desiccated on plastic or stainless steel as described in Fig. 1. Following incubation periods of 1, 3, or 7 days, cells were recovered and genomic DNA was isolated for library preparation and next-generation sequencing. Cells that were not subjected to desiccation (time zero) served as the input control (NA). The input control had >60,000 unique insertion mutants. Plotted is the percentage of unique insertion mutants recovered at each time point and from each surface. The Tn-seq results are from 2 biological replicates.

The first Tn-seq experiment was exploratory in nature and performed by diluting an aliquot of the mutant library in PBS and spotting 6 × 10^8^ cells onto the surface of plastic petri dishes or stainless-steel discs in duplicate (Fig. S1). The surfaces were desiccated in a biological hood for 3 h under laminar flow conditions and then incubated at room temperature for 1, 3, or 7 days. Surviving cells were recovered in PBS and allowed to outgrow to an OD_600_ of 0.5 in fresh LB media. Although quantitation of the recovered cells was not performed directly, the time required for the recovered cells to reach an OD_600_ of 0.5 was directly correlated with the length of the desiccation period, ranging from 12 h following 1 day of desiccation to 18 h for the day 7 samples (Table S2). The latter finding is consistent with the findings in Fig. 1 where loss of viability was directly correlated with desiccation time. The two biological replicates provided at least 14 million sequence reads for each condition (Table S1A). Pearson correlation coefficients (R^2^) for unique insertions and reads per insertion were 0.8 and 0.83, respectively (Fig. S2). There was a progressive loss of unique insertion mutants recovered from plastic and steel surfaces that correlated with the length of the desiccation period. Compared to the input sample, 70% of the unique insertions were recovered from plastic following 1 day of desiccation, 30% after 3 days, and 14% after 7 days (Fig. 2). The findings were similar on the steel surface with 80% recovery on day 1, 23% after 3 days, and 11% on day 7. There were no significant differences between the total number of mutants that survived desiccation on plastic and on steel at each time point.

10.1128/msystems.00114-22.1 FIG S1 Schematic of the first Tn-seq experiment. The PA14 Tn-seq library was diluted to 3 × 10^8^ cell/mL in PBS, and 40-μL aliquots (2 mL total) were spotted on plastic or stainless-steel surfaces, desiccated under ambient conditions, and incubated for 1, 3, or 7 days. Cells were recovered by hydrating the surface and dislodging cells and then cultured in LB broth to an OD_600_ of 0.5. The control/input for the experiment was not subjected to desiccation. Tn-seq was performed on the input and output libraries. Download FIG S1, TIF file, 2.3 MB. Copyright © 2022 Karash and Yahr. 2022 Karash and Yahr. https://creativecommons.org/licenses/by/4.0/ This content is distributed under the terms of the Creative Commons Attribution 4.0 International license.

10.1128/msystems.00114-22.2 FIG S2 Tn-seq experiment reproducibility. Correlation between unique insertions per ORF (A) and the number of reads per insertion (B) for the nondesiccated input-1 and input-2 control samples. Download FIG S2, TIF file, 1.6 MB. Copyright © 2022 Karash and Yahr. 2022 Karash and Yahr. https://creativecommons.org/licenses/by/4.0/ This content is distributed under the terms of the Creative Commons Attribution 4.0 International license.

10.1128/msystems.00114-22.9 TABLE S2 Time required for cells recovered from plastic (P) or stainless-steel (S) surfaces to reach culture OD_600_ of 0.5 following 1-, 3-, or 7-day (D) desiccation periods with two replicates (rep 1 or 2). Download Table S2, TIF file, 1.9 MB. Copyright © 2022 Karash and Yahr. 2022 Karash and Yahr. https://creativecommons.org/licenses/by/4.0/ This content is distributed under the terms of the Creative Commons Attribution 4.0 International license.

We postulated that unrecovered/underrepresented mutants have insertions that impair survival on desiccated surfaces. Insertion mutants lost due to desiccation were considered important for desiccation tolerance (Data set 1). From these analyses, we identified 119 genes at D1, 548 at D3, and 1,819 at D7 that contribute to desiccation tolerance on a plastic surface and 100, 886, and 1,478 genes important for survival on stainless steel. On the plastic surface, 70 (59%) of the genes identified on D1 were also present on D3, and 98 (82%) genes were present on D7. Likewise, 424 (77%) of the genes identified on D3 were also present in D7 (Fig. S3A). The findings were similar for the steel surface wherein 75 (75%) and 66 (66%) genes from D1 were present in the D3 and D7 samples, respectively, and 586 (66%) genes from D3 were also found on D7 (Fig. S3B). Comparison of the findings from plastic to steel revealed that 38 (32%) genes were common between plastic and steel at D1, 325 (59%) at D3, and 955 (53%) at D7 (Fig. S3C). The finding that many of the genes important for P. aeruginosa survival are shared at the various time points on plastic and steel surfaces suggests nonstochastic depletion of the mutants. While only 32% of the genes were common between plastic and steel on D1, there was significant overlap in the biological processes represented by the complete gene sets identified under each condition (Table S3). Those processes included metabolism of amino acids, carbohydrates, lipids, purine and pyrimidine, cofactors, and outer membrane biosynthesis. Because the cells were initially suspended and desiccated in PBS, we speculated that reduced viability could reflect nutritional stress, desiccation stress, or a combination of both.

10.1128/msystems.00114-22.3 FIG S3 Consensus P. aeruginosa genes important for desiccation tolerance on plastic and stainless-steel surfaces. Number of genes important for desiccation tolerance at 1, 3, or 7 days (D) desiccation identified by Tn-seq on plastic (A) and stainless-steel (B) surfaces. (C) Consensus genes shared between the plastic and stainless surfaces. Download FIG S3, TIF file, 1.1 MB. Copyright © 2022 Karash and Yahr. 2022 Karash and Yahr. https://creativecommons.org/licenses/by/4.0/ This content is distributed under the terms of the Creative Commons Attribution 4.0 International license.

10.1128/msystems.00114-22.10 TABLE S3 Pathways important for desiccation tolerance on plastic and steel surfaces from the first Tn-seq experiment. Download Table S3, TIF file, 2.4 MB. Copyright © 2022 Karash and Yahr. 2022 Karash and Yahr. https://creativecommons.org/licenses/by/4.0/ This content is distributed under the terms of the Creative Commons Attribution 4.0 International license.

### Distinguishing viability loss resulting from nutritional and/or desiccation stresses.

**FIG 3:**
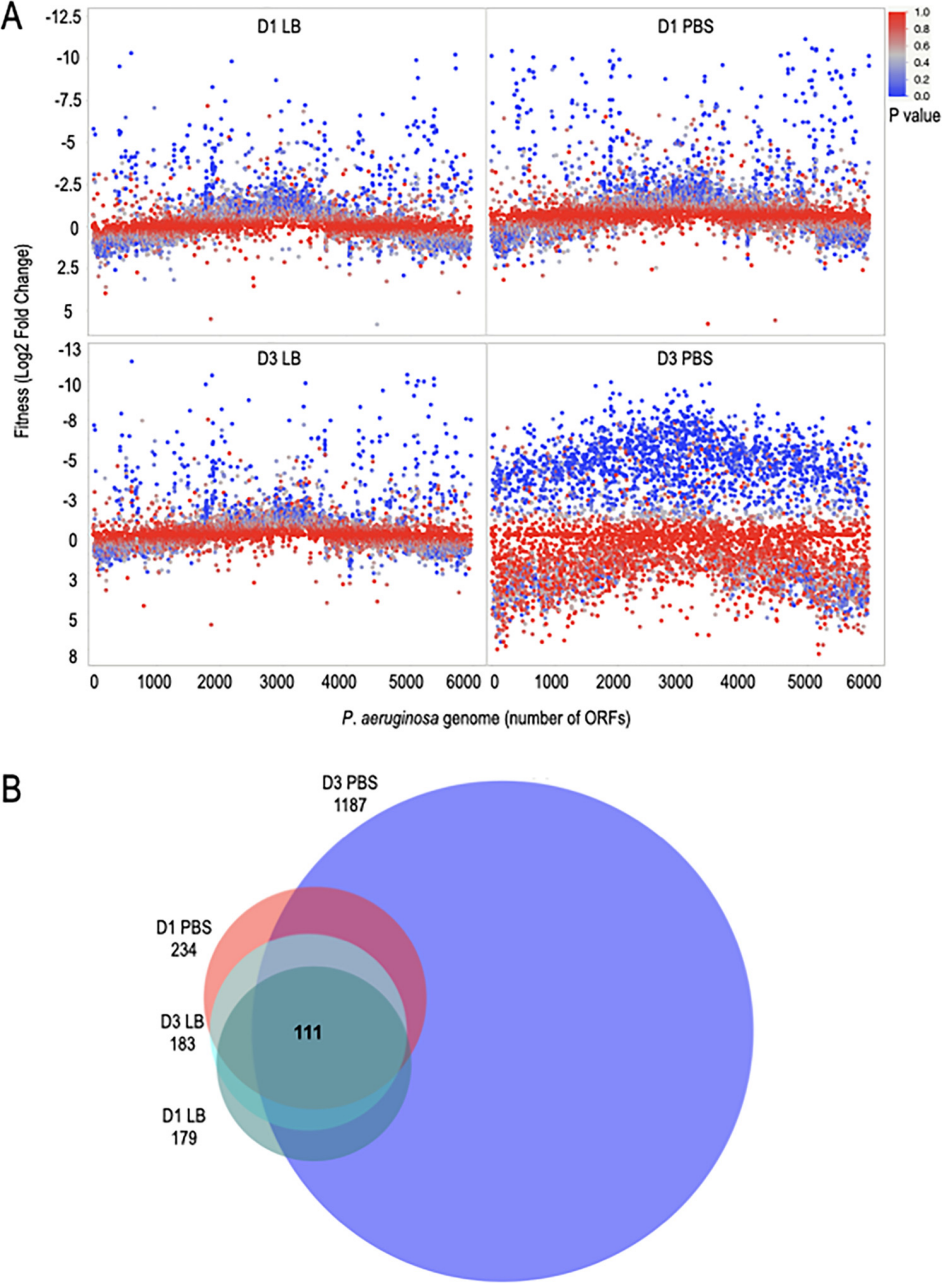
Desiccated P. aeruginosa genome mutant fitness in the presence (LB) and absence (PBS) of nutrients. (A and B) The transposon mutant library was desiccated on plastic in presence (LB broth) or absence (PBS) of nutrients. Cells were recovered 1 or 3 days (D) postdesiccation, and genomic DNA and libraries were prepared for sequencing. Cells that were not subjected to desiccation served as the input control. The input library had 88,425 unique insertions. (A) Plotted is the log_2_ fold change in reads after desiccation compared to the control for each P. aeruginosa ORF (open reading frame). The Tn-seq results are from 2 biological replicates. (B) Venn diagram showing the number of genes that had a log_2_ fold change of <−2 and an adjusted *P* value of <0.05 for LB or PBS at day 1 or day 3. There were 111 consensus genes shared in the 4 conditions tested. The Venn diagram was generated by DeepVenn (59).

To differentiate genes sensitive to desiccation in PBS from those truly important for desiccation tolerance, we performed another Tn-seq experiment wherein the transposon mutant library was desiccated on plastic in the absence (PBS) and presence (LB) of nutrients for either 1 or 3 days with two biological replicates for each condition (Fig. S4 and Table S1B). Viable cells were recovered by rehydrating the surfaces under the same conditions used for desiccation (e.g., rehydrated in PBS if desiccated in PBS) and plating on Vogel-Bonner medium (VBM) agar plates. Viable cells were collected after 24 h, and genomic DNA was prepped for NexGen sequencing. The two biological replicates provided at least 80 million sequence reads per condition, and the mean number of reads was 435 per TA insertion site (Table S1B). Pearson correlation coefficients (R^2^) for unique insertions of the nondesiccated inputs and D1 (desiccation day 1, D1) conditions were 0.99, 0.98, and 0.97, respectively (Fig. S5A to C). The R^2^ for unnormalized reads per insertion of nondesiccated conditions was 0.94; this value was raised to 0.98 with normalization (Fig. S5D to E). Gene fitness was calculated by comparing reads from the input controls to the recovered samples and considered significant if the log_2_ fold change was at least −2 with an adjusted *P* value of <0.05 (Fig. 3A, Data set 2). Samples harvested on D1 revealed 234 and 179 genes with fitness defects in the PBS and LB samples, respectively. By D3, the number of genes with impaired fitness reached 1,187 for PBS but remained similar for LB at 183. The selective reduction in fitness for the PBS sample on D3 suggested that examination of the consensus gene sets could be used to distinguish genes important for desiccation tolerance from those lost due to nutritional stress. There were 141 shared genes on D1, 149 on D3, and 111 common to both D1 and D3 when desiccated in PBS or LB (Fig. 3B). The remainder of our analyses focused on these 111 genes.

10.1128/msystems.00114-22.4 FIG S4 Schematic of the second Tn-seq experimental. The PA14 Tn-seq library was diluted to 1.5 × 10^9^ cells/mL in PBS or LB, and 40-μL aliquots (2 mL total) were spotted on a plastic surface, desiccated under ambient conditions, and incubated for 1 or 3 days. Cells were recovered in PBS or LB and plated on VBM agar plates. Cells were collected from the plates and used for Tn-seq library preparation. The control/input for the experiment was not subjected to desiccation. Tn-seq was conducted on the input and output libraries. Download FIG S4, TIF file, 1.1 MB. Copyright © 2022 Karash and Yahr. 2022 Karash and Yahr. https://creativecommons.org/licenses/by/4.0/ This content is distributed under the terms of the Creative Commons Attribution 4.0 International license.

10.1128/msystems.00114-22.5 FIG S5 Tn-seq experiments were highly reproducible. (A) Correlation of unique insertions per ORF of the nondesiccated input-1 and input-2 controls. (B and C) Unique insertions per ORF for LB (B) and PBS (C) at day 1 (C). (D and E) Number of nonnormalized (D) and normalized (E) reads per insertions for input-1 and input-2. Download FIG S5, TIF file, 1.4 MB. Copyright © 2022 Karash and Yahr. 2022 Karash and Yahr. https://creativecommons.org/licenses/by/4.0/ This content is distributed under the terms of the Creative Commons Attribution 4.0 International license.

To validate the Tn-seq findings, competition assays were performed with wild-type (WT) P. aeruginosa and select transposon insertion mutants within genes demonstrating a fitness defect. The transposon mutants, marked with a gentamicin resistance cassette, were obtained from the P. aeruginosa PA14 transposon library (27) and verified by PCR amplification of the transposon junction and DNA sequencing. Strains were cultured in LB to stationary phase, washed in PBS, mixed 1:1 (10^8^ cells each of WT and a Tn mutant), and allowed to desiccate on plastic. The next day, the cells were recovered in LB, serially diluted, and plated on LB and LB agar plates containing gentamicin to determine a competitive index (CI). We first tested mutants in 5 of the 14 genes that had fitness changes in the 4- to 8-fold range at D1 when desiccated in PBS. Experiments with these 5 mutants revealed no significant change in the CI (Fig. S6A).

10.1128/msystems.00114-22.6 FIG S6 (A) Genes with fitness defects greater than log_2_ −3 are not important for desiccation tolerance. Transposon insertion mutants with log_2_ fold changes greater than −3 in the Tn-seq experiment were competed with WT cells and desiccated on plastic for 1 day. None of the tested mutants showed a significant reduction in the CI. (B) Tn-seq profile of *ostA-surA-pdxA* operon. The profile shows number of TA sites, unique insertions, and reads associated with each insertion for the input control and day 1 following desiccation on plastic. Download FIG S6, TIF file, 0.6 MB. Copyright © 2022 Karash and Yahr. 2022 Karash and Yahr. https://creativecommons.org/licenses/by/4.0/ This content is distributed under the terms of the Creative Commons Attribution 4.0 International license.

**FIG 4:**
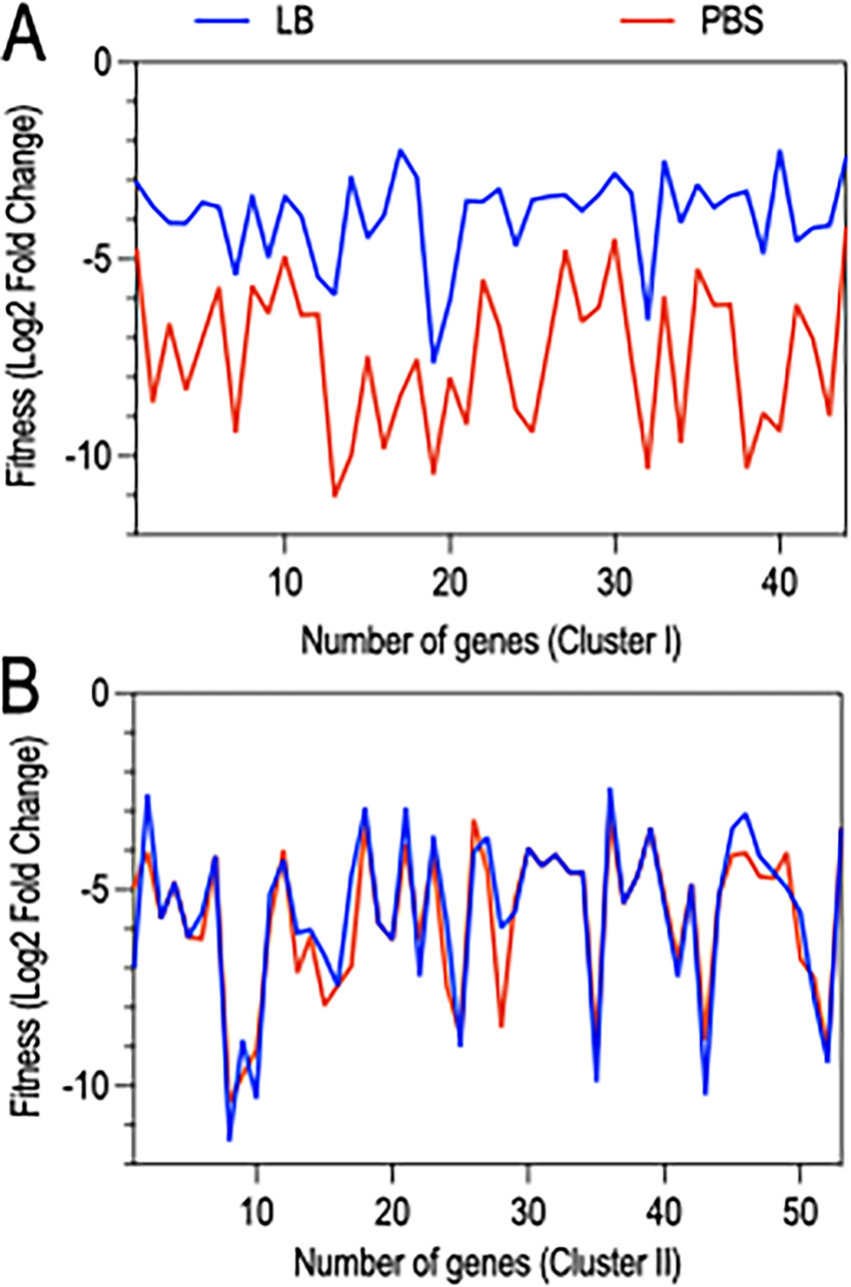
Differentiating genes required for desiccation and/or nutritional tolerance from those specific to desiccation tolerance. (A and B) Plotted is the log_2_ fold change for cluster I (A) and cluster (II) (B) genes desiccated on plastic in the presence (LB) or absence (PBS) of nutrients and recovered after a 1-day incubation period with an adjusted *P* value of <0.05. (A) The fitness of cluster I genes (44 in total) is reduced in the absence of nutrients (PBS) compared to that in the presence of nutrients (LB broth), suggesting that they are required for desiccation tolerance and nutritional stress. (B) The fitness of cluster II genes (53 genes in total) is similar in the absence (PBS) and presence (LB broth) of nutrients, indicating that they are required for desiccation tolerance only.

**FIG 5:**
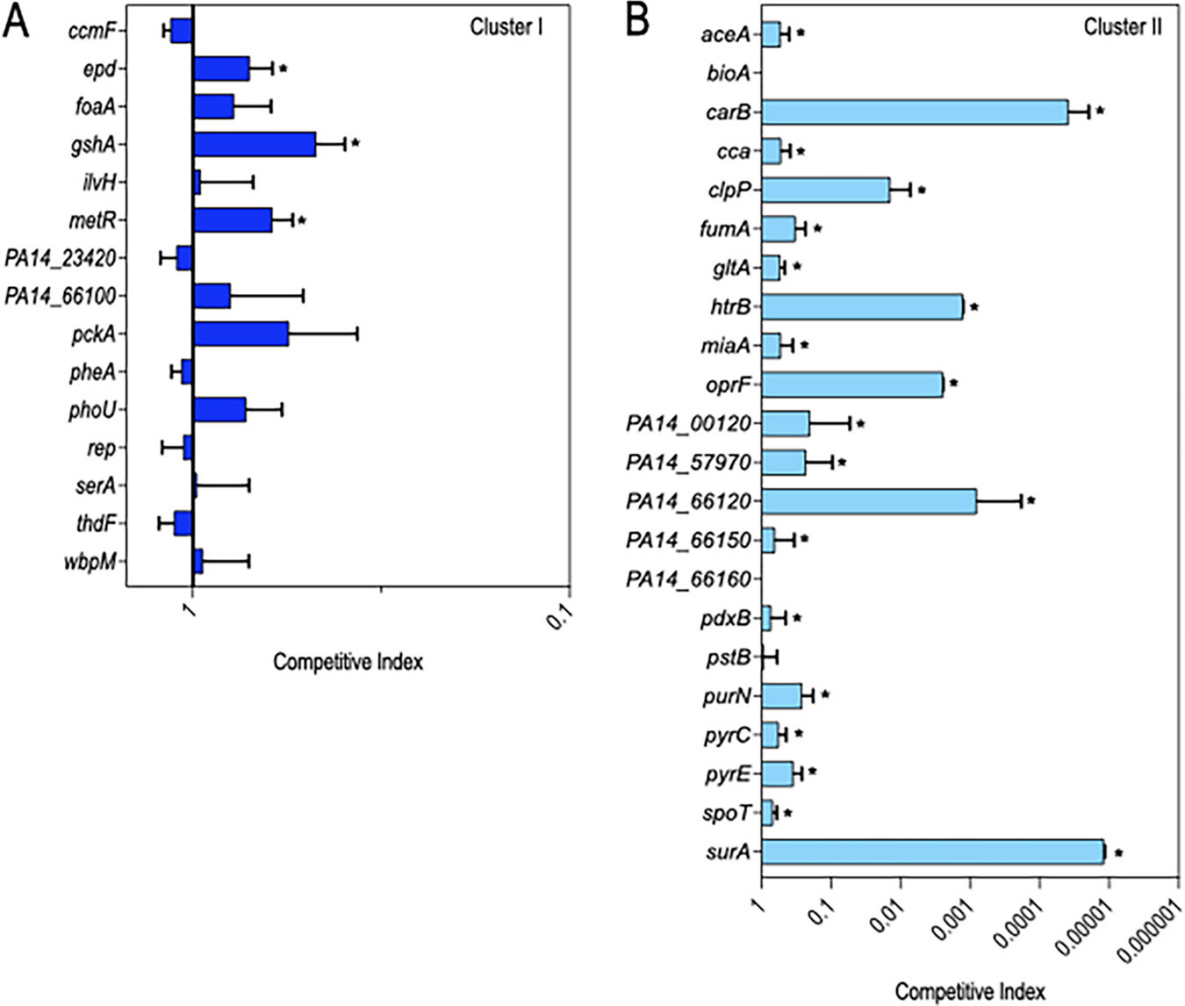
Competition assays to validate genes important for desiccation tolerance. (A and B) Stationary phase cultures of WT cells and individual transposon insertion mutants (marked with a gentamicin resistance cassette) were suspended in PBS, mixed 1:1 (10^8^ cells of each strain), desiccated on plastic, and incubated at room temperature. The next day, cells were recovered and plated on LB and LB plates containing gentamicin to determine a competitive index. The competitive indices for 15 of the cluster I genes (A) with roles in nutritional stress and/or desiccation tolerance and 22 of the cluster II genes (B) important for desiccation tolerance. An arbitrary number was assigned for *carB* and *surA* to calculate CI, as no viable cells were recovered for these 2 mutants. Data represent the average of at least 3 replicates. Mutants with significant reductions in the CI are indicated with an asterisk (*P* < 0.05).

We thus focused on the remaining 97 genes, which had >8-fold fitness changes (Data set 3) when desiccated in PBS at D1. The 97 genes were categorized into two clusters based on a comparison of the fitness values from the PBS and LB conditions. Cluster I genes (*n* = 44) had a larger fitness defect when desiccated in PBS compared to that when desiccated in LB at D1 (Fig. 4A). This observation suggested that cluster I genes are sensitive to the effects of desiccation (as evidenced by decreased fitness in LB) and that the defect is further exacerbated by desiccation in PBS. It was thus surprising that competition assays performed on 15 of the cluster I genes revealed no significant effect on the CI for 12 genes, with values ranging from 0.5 to 1.2 (Fig. 5A). A key difference between the second Tn-seq selection and the competition experiments was the method used to recover cells following desiccation. For the Tn-seq experiment, the surviving cells were suspended and recovered on VBM agar plates while survivors from the competition experiments were suspended and recovered on LB agar plates. The Tn-seq conditions, therefore, were more stringent and identified genes important for survival under both low-nutrient conditions and desiccation, while the competition experiments were less stringent and more specific to desiccation tolerance.

Cluster II genes (*n* = 53) were defined as having similar fitness defects in both LB and PBS at D1 (Fig. 4B). Many of the cluster II genes were not recovered at all or only in low abundance from LB and PBS on D1 (Data set 3). Competition assays with mutants lacking 22 of the cluster II genes revealed that 19 had reduced fitness (Fig. 5B). Experiments with the *carB* and *surA* strains resulted in the recovery of no mutant cells, and *clpP*, *htrB*, *oprF*, *PA14_00120*, *PA14_57970*, and *PA14_66120* mutants exhibited severe reductions in the competitive index (CI).

### The reduced fitness of cluster II genes reflects desiccation intolerance.

**FIG 6:**
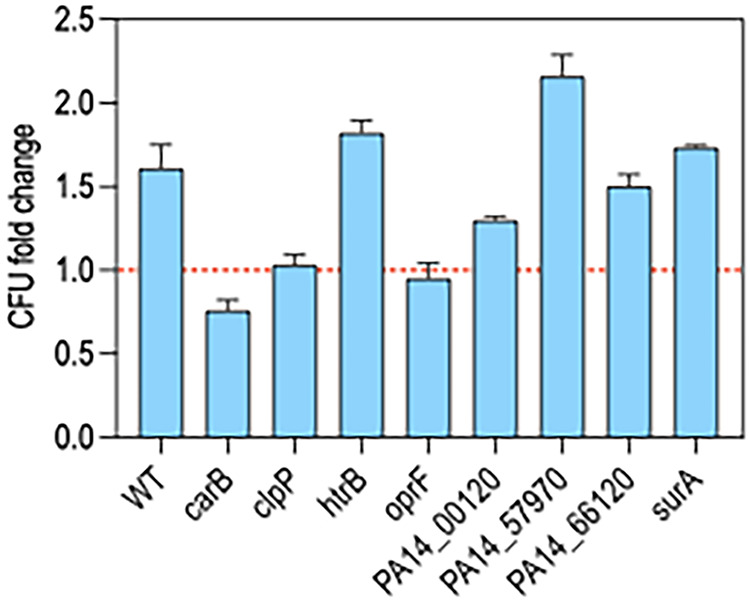
Mutants lacking genes required for desiccation tolerance are not starvation sensitive. Transposon mutants lacking Cluster II genes that demonstrated significant defects in the CI (Fig. 5) were tested for starvation survival. Cells were cultured overnight in LB, washed twice in PBS, suspended in PBS to 10^8^ cells/mL, and incubated in a flask at room temperature under static conditions. Cells were collected at time zero (before starvation) and after 1 day, enumerated by serial dilution, and plated on LB agar plates. Plotted is the fold change in CFU following starvation for 1 day.

We considered a number of trivial explanations to account for the reduced fitness of cluster II genes. The Tn-seq selection for low-nutrient conditions was performed by washing and desiccating cells in PBS. To determine whether loss of fitness was triggered by exposure to PBS, cluster II mutants with the largest fitness defects were cultured to stationary phase, suspended in PBS, and incubated at room temperature for 24 h. Cells were enumerated by serial plating on LB and compared to the number of cells present at time zero. There was a modest increase in the number of WT cells and the *PA14_57970*, *PA14_66120*, *PA14_00120*, *htrB*, and *surA* mutants following the 24-h incubation period (Fig. 6). The *oprF* and *clpP* mutants demonstrated no increase in cell numbers and no significant loss in viability. Only the *carB* mutant had a small reduction in viability (25%) following 24 h (Fig. 6). Given the significant fitness defects observed following desiccation and recovery, however, we conclude that simple exposure to PBS was not a significant contributing factor to fitness loss.

Irrespective of being desiccated in PBS or LB, the surviving cells were recovered by plating on VBM agar. To determine whether reduced fitness reflected an inability to grow on VBM, the 37 individual cluster I and II mutants used in the competition experiments were tested for growth on VBM agar plates. Seven of the 42 mutants were unable to grow and are presumably auxotrophs (Data set 3). Extrapolating that rate to the entire collection of 97 genes suggests that ~17% are auxotrophs. Cluster I genes had 3 auxotrophs out of 15 mutants, and cluster II had 4 auxotrophs (*carB*, *pyrC*, *pyrE*, and *spoT*) out of the 22 tested mutants (Data set 3). Competition and survival assays show that *carB* mutants cannot tolerate desiccation under the conditions of our experiments and *pyrC*, *pyrE*, and *spoT* exhibited a significant reduction of CI (Fig. 5B). Conversely, the 4 cluster II mutants (*bioA*, *PA14_66160*, *pdxB*, and *pstB*) with no defect in the CI were not auxotrophs on VBM (Data set 3). This suggests that the probability of false-positive genes in the list of genes due to auxotrophy is small based on the phenotype confirmation of 38% of genes.

**FIG 7:**
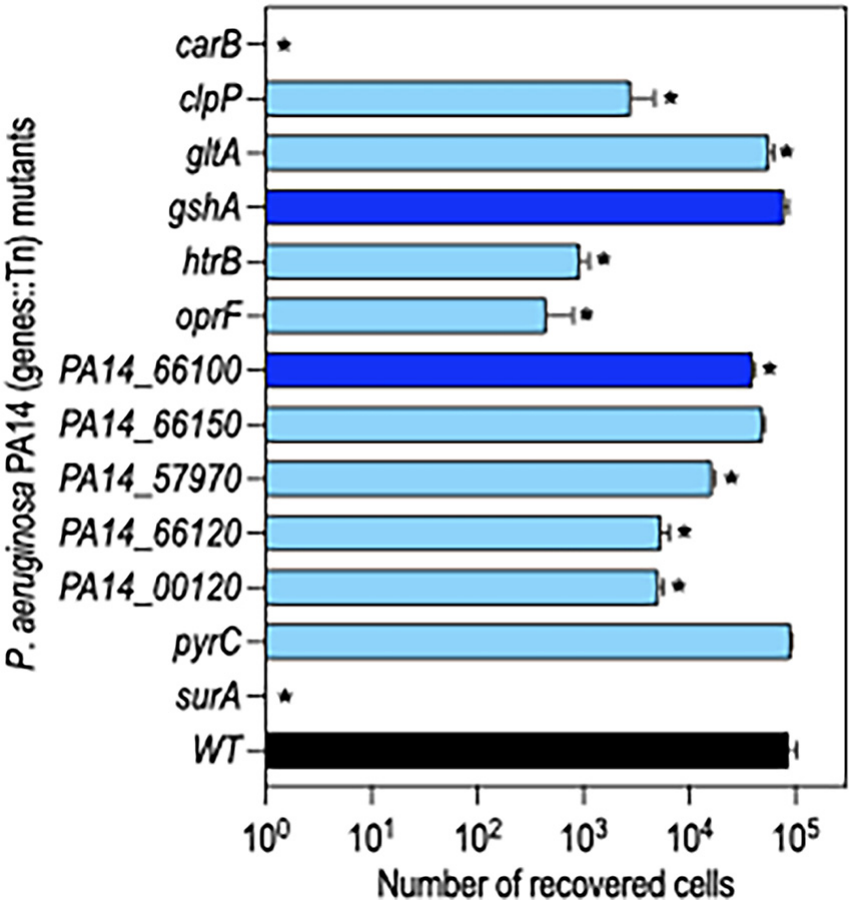
Survival of select cluster I and II mutants when desiccated alone. WT cells (black bar) and the indicated transposon insertion mutants with cluster I (dark blue bars) and cluster II (light blue bars) genes were cultured to stationary phase, washed with PBS, and 10^8^ cells were desiccated on plastic. The next day, cells were recovered and enumerated by serial dilution and plating. Plotted in the number of CFU recovered for each strain. No cells were recovered for *carB* and *surA* mutants. Mutants with significant reductions in recovery relative to WT cells are indicated with an asterisk (*P* < 0.05). Data represent the average of at least 3 replicates.

We also considered bottleneck effects since the Tn-seq selections were conducted by competing a library of Tn insertion mutants simultaneously and the competition experiments were performed by competing single mutants with an equal number of WT bacteria. To determine the fitness of the mutants in isolation, 2 of the cluster I mutants and 11 of the cluster II mutants were desiccated individually in the absence of WT cells. Consistent with Tn-seq results and competition assays, most of the cluster II mutants had reduced tolerance to desiccation (Fig. 7). None of the 10^8^ desiccated *carB* and *surA* mutant cells, and <1,000 of the *htrB* and *oprF* cells, were recovered after 24 h. The other mutants showed comparable survival rates to the competition assay results and Tn-seq profiles (Fig. 5). We conclude that many, but not all, cluster II genes contribute to desiccation tolerance.

## DISCUSSION

In this study, we systematically screened P. aeruginosa for genes required for desiccation tolerance and survival. Whereas cells grown to exponential phase had poor tolerance to desiccation, stationary-phase-grown cells tolerated desiccation on plastic and stainless-steel surfaces for at least 7 days (Fig. 1). The observation that the physiological state of the cells is critical to desiccation tolerance is consistent with previous studies using P. aeruginosa and other bacteria (28, 29). Although stationary-phase cells tolerated desiccation, there was significant attrition in the desiccated population, with only 0.05 to 0.1% of the cells surviving 24 h postdesiccation. We were initially concerned that the surviving cells were suppressor mutants with increased desiccation tolerance and/or that the decline in viability reflected stochastic loss. Both concerns were unfounded. Cells recovered from a typical desiccation assay and then desiccated a second time were recovered at the same rate (Fig. 1B), indicating that there is no immediate fitness advantage conferred by surviving desiccation. Likewise, our finding of significant overlap in the unique insertions lost on D1, D3, and D7 for both the plastic and steel surfaces was consistent with nonstochastic death (Fig. S3A to B). The high attrition that we observed likely reflects the stringency of the experimental conditions. In addition to the physiological state of the cells prior to desiccation, several other factors influence survival rates. Prolonging the desiccation period and slowing the rehydration rate both increase survival by providing cells an opportunity to induce protective responses and by reducing the effects of osmotic and oxidative stresses (30).

Because the initial Tn-seq experiment was performed by desiccating cells in PBS, we were unable to differentiate genes important for surviving nutritional stress from those required for desiccation tolerance. Performing a second Tn-seq experiment where cells desiccated in PBS were compared to cells desiccated in LB led to the identification of cluster II genes, defined by reduced fitness under both desiccation conditions (PBS and LB). The list consisted of 53 genes enriched in several pathways, including outer membrane biogenesis genes, LPS modification, purine and pyrimidine biosynthesis, tricarboxylic acid cycle, tRNA processing, amino acid biosynthesis, hydrolysis of proteins, and a few uncharacterized genes. We highlight some of these genes and discuss potential roles in desiccation tolerance.

**FIG 8:**
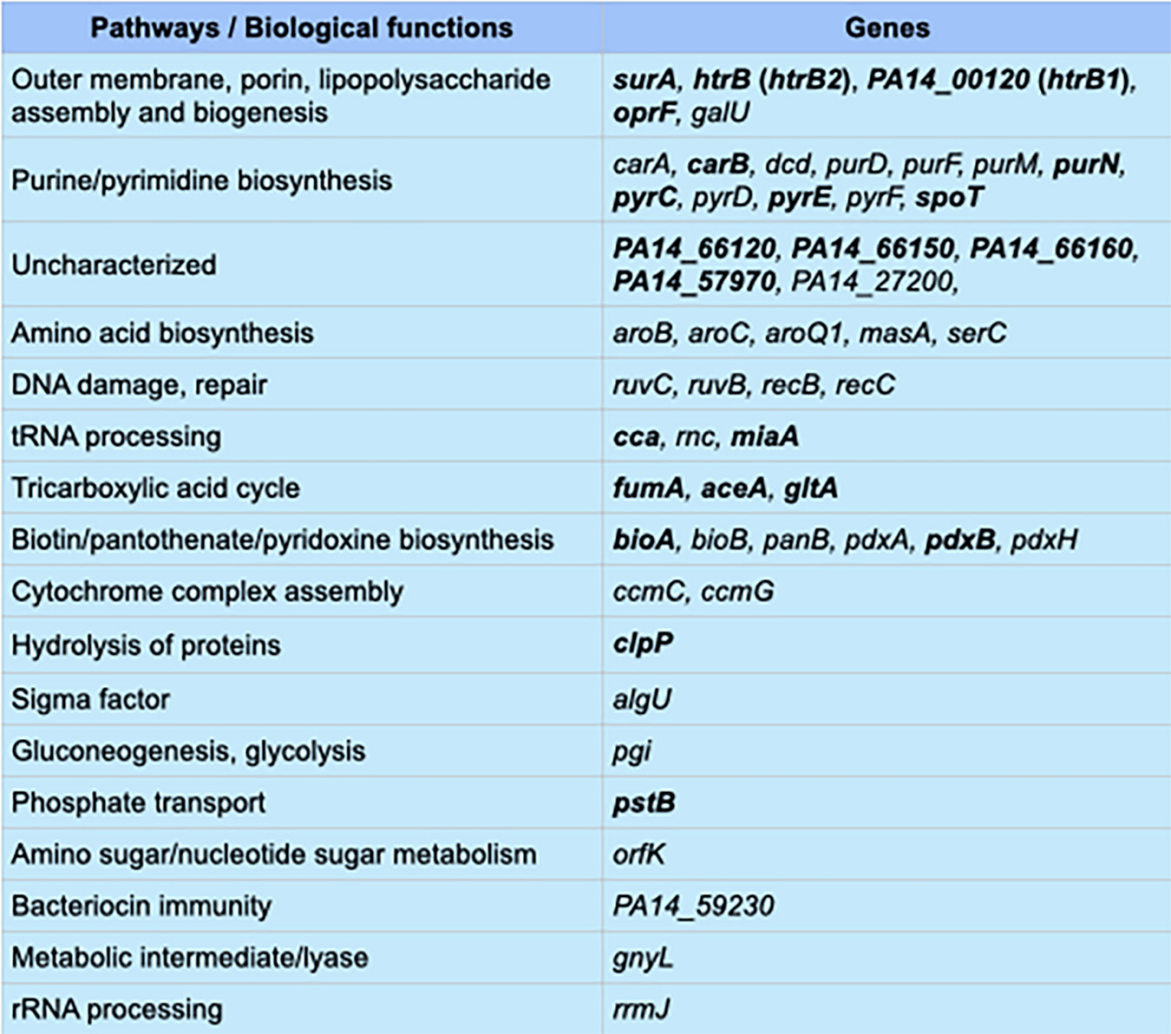
Genes and pathways important for desiccation tolerance. Genes with reduced fitness (log_2_ fold change < −3) and adjusted *P* values of <0.05 when desiccated in PBS and LB. Bold genes represent individual mutants used in the competition assays shown in Fig. 5 and Fig. S6A.

One category of genes important for desiccation tolerance was related to the cell envelope and included *surA*, *oprF*, *htrB*, *PA14_00120*, and *galU* (Fig. 8). Escherichia coli SurA is a multifunctional protein with peptidyl-prolyl isomerase and chaperone activities, both of which contribute to the biogenesis of outer membrane proteins (OMP) (31, 32). Depletion of P. aeruginosa SurA alters outer membrane protein (OMP) integrity and composition, increases sensitivity to antibiotics, and attenuates virulence in an infection model (33). SurA is encoded in a 3-gene operon (*ostA-surA-pdxA*). Our Tn-seq data indicate that *ostA* is an essential gene while *surA* and *pdxA* are nonessential (Fig. S6B) (34). Whether *surA* is essential, however, is an open question and could be strain dependent. Some studies indicate that transposon insertions in *surA* are not tolerated (35, 36), while others, including this study, suggest otherwise (27). The *surA* mutant tested in the competition assay is disrupted by a transposon insertion at codon number 6. To confirm that the desiccation phenotype resulted from *surA* deficiency, the *surA*::Tn mutant was transformed with a vector control or a *surA* expression vector and tested for desiccation tolerance. Similar number of cells were recovered 24 h postdesiccation from WT cells harboring either vector and for the *surA*::Tn mutant complemented with *surA* (Fig. S7A). The *surA*::Tn mutant harboring the vector control, however, was completely defective in desiccation tolerance. These results confirm that *surA* is required for desiccation tolerance. Given the importance of SurA to OMP biogenesis, it seems likely that the defect in desiccation tolerance is pleotropic and results from effects on numerous OMPs. One that may be particularly important is OprF, a major outer membrane porin required for cell structure and outer membrane permeability (37). In our competition experiments, the *oprF*::Tn mutant had a significantly reduced CI (Fig. 5B) and when desiccated alone demonstrated a 192-fold reduction in tolerance compared to that of WT (Fig. 7). The tolerance defect in the *oprF* mutant, while severe, was not to the level of the *surA*::Tn defect. This suggests that additional SurA-dependent factors contribute to the phenotype of the *surA* mutant.

10.1128/msystems.00114-22.7 FIG S7 Phenotype validation of *surA*, *relA spoT*, *carB*, and *rpoS* mutants. (A) WT strain PA14 and *surA*::Tn carrying either a vector control (pJN) or a *surA* expression vector (pSurA) were cultured to stationary phase. Cells (10^8^) were desiccated on plastic for 1 day and then recovered, serial diluted, and plated on LB agar. Plotted is the number of CFU recovered for each strain. (B) WT strain PAO1 and a *relAspoT* mutant were desiccated as described above. Plotted is the number of CFU recovered for each strain. (C) WT strain PA14 was cultured to stationary phase in LB, and *carB*::Tn cells were cultured to stationary phase in LB broth supplemented with 2.5 mM uracil and/or 3 mM l-arginine as indicated. Cells were mixed 1:1 (10^8^ cells of each strain), desiccated on plastic, and incubated at room temperature. The next day, cells were recovered and plated on LB and LB plates containing gentamicin to determine a competitive index. (D and E) WT strain PA14 and an *rpoS*::Tn mutant were cultured to stationary phase. Cells (10^8^) were desiccated on plastic for 1 day alone (panel D) or together (1:1 ratio) and then recovered, serial diluted, and plated on LB agar. Plotted is the number of CFU recovered for each strain in panel D and the competitive index in panel E. Download FIG S7, TIF file, 1.7 MB. Copyright © 2022 Karash and Yahr. 2022 Karash and Yahr. https://creativecommons.org/licenses/by/4.0/ This content is distributed under the terms of the Creative Commons Attribution 4.0 International license.

The remaining three envelope-related genes (*galU*, *htrB*, and *PA14_00120*) with reduced tolerance to desiccation are involved in lipopolysaccharide (LPS) biosynthesis. The *galU* gene encodes UTP glucose-1-phosphate uridylyltransferase and participates in LPS core biosynthesis by catalyzing UDP-glucose from glucose-1-P (38). *htrB* and *PA14_00120* encode predicted acyltransferases, HtrB2 and HtrB1, respectively. Bacteria use these enzymes to remodel the lipid A portion of LPS in response to environmental signals. HtrB2 adds laurate to tetra-acylated lipid A to generate a penta-acylated form, and HtrB1 adds 2-hydroxylaurate to form hexa-acylated lipid A (39). Several studies in other organisms report a role for lipid A remodeling in desiccation tolerance. In Rhizobium leguminosarum, addition of 27-hydroxyoctacosanoic acid to the 2′ position of lipid A improves tolerance to desiccation (40), and in Acinetobacter baumannii, hexa-acylated lipid A promotes tolerance while penta-acylation confers extreme sensitivity (41). In the latter case, the authors proposed that hexa-acylated lipid A reduces membrane fluidity and enhances retention of water and nutrients. The CI of the *htrB* mutant was close to zero and when desiccated alone demonstrated a fitness 93-fold lower than that of WT (Fig. 5B and Fig. 7). The CI of the *PA14_00120* mutant was less severe (0.2), as was the fitness defect (17-fold), when desiccated alone. These findings suggest that hexa-acylated lipid A is critical for desiccation tolerance in P. aeruginosa.

Several stress response pathways important for cell envelope homeostasis also demonstrated a role in desiccation tolerance. ClpP is a caseinolytic peptidase which plays a central role in the degradation of misfolded proteins (42). The CI of the *clpP*::Tn mutant was 0.01, and when desiccated alone, there was a 31-fold decrease in recovery of the mutant (Fig. 5B and Fig. 7). ClpP positively influences biofilm formation, motility, and alginate biosynthesis (43, 44). Alginate production was of particular interest given the prior association with desiccation tolerance (19) and our identification of *algU* in the Tn-seq screen. AlgU is an extracytoplasmic function sigma factor and a key activator of alginate production (45). None of the genes directly involved in alginate biosynthesis, however, demonstrated a fitness defect. We offer a few explanations for this finding. First, the experimental conditions (limited in both time and energetic resources) used in our studies likely precluded the biosynthesis of alginate, which is energetically expensive, on a scale sufficient to promote desiccation tolerance. Second, the AlgU regulon is extensive (372 genes) and includes many genes important for stress responses and cell envelope homeostasis. The AlgU requirement for desiccation tolerance, therefore, is likely pleiotropic in nature and relies upon numerous factors. Thus, small contributions by some of those factors may not be revealed by the Tn-seq approach.

The stringent response is an adaptation to nutrient-limiting environments. In response to starvation, RelA and SpoT synthesize guanosine 3′,5′-bispyrophosphate [ppGpp(p)]. SpoT can also degrade ppGpp (46, 47). ppGpp(p) interacts with RNA polymerase, reduces transcription of rRNA and structural components of the ribosome, and enhances transcription of biosynthetic genes, including those required for amino acids. Both the Tn-seq and competition experiment supported a role for *spoT* in desiccation tolerance (Fig. 5B). To further validate that finding, we tested a strain PAO1 Δ*relA spoT* mutant in the competition assay. Compared to WT PAO1, there was a 12-fold reduction in the recovery of Δ*relA spoT* mutant cells (Fig. S7B). These data suggest that the stringent response plays a role in desiccation tolerance and is consistent with our identification of amino acid biosynthetic genes in the Tn-seq screen. In fact, nearly half of cluster I genes belong to amino acid biosynthetic pathways (Data set 3). Given the extensive nature of the stringent response, its involvement in desiccation tolerance is likely pleiotropic and involves genes that extend beyond amino acid biosynthetic pathways.

We also identified roles for pyrimidine (*carAB*, *pyrCDEF*, and *dcd*) and purine (*purDFMN*) biosynthetic genes in desiccation tolerance (Fig. 8). The *carAB* genes encode the small and large subunits of carbamoyl-phosphate synthetase, respectively. Carbamoyl-phosphate synthetase catalyzes the ATP-dependent synthesis of carbamoyl phosphate, a precursor for both pyrimidine and arginine biosynthesis (48). The *carB*::Tn mutant was unable to tolerate desiccation competed with WT or desiccated alone (Fig. 5B and Fig. 7). We noticed that overnight cultures of the *carB*::Tn mutant had lower growth yields in LB (~OD_600_ of 1.0) relative to those of WT cells (~OD_600_ of 2.2) (Data set 3). Our finding that desiccation tolerance is dependent upon prior growth to stationary phase suggested that the *carB*::Tn mutant phenotype might result from an alteration in stationary-phase growth. Culturing the *carB*::Tn mutant overnight in LB supplemented with either uracil or uracil and arginine resulted in growth yields similar to those of WT cells. When cultured under those conditions, the *carB*::Tn mutant effectively competed with WT cells (CI of ~1) (Fig. S7C). No viable cells were recovered using *carB*:Tn cells grown in medium lacking uracil and arginine. This finding demonstrates that *carB* is required for desiccation tolerance, probably owing to a role in achieving an appropriate physiologic state upon reaching stationary phase. Several genes in the purine biosynthetic pathway were also identified by Tn-seq. The CI values for *purN*::Tn, *pyrC*::Tn, and *pyrE*::Tn mutants were 0.3, 0.6, and 0.3, respectively (Fig. 5B). While those values are fairly modest, they collectively support purine biosynthetic playing some role in desiccation tolerance.

Given that growth to stationary phase was required for desiccation tolerance, we were surprised that the stationary phase-associated sigma factor (*rpoS*) was not identified in the Tn-seq screen. To confirm that *rpoS* is not important for desiccation tolerance under the conditions of our screen (an important stipulation), a PA14 *rpoS*::Tn transposon insertion mutant was tested for desiccation tolerance alone and in competition with WT cells. Consistent with our Tn-seq results, the number of *rpoS*::Tn cells desiccated alone and then recovered after 24 h was similar to that of WT cells (Fig. S7D). Likewise, similar numbers of WT and *rpoS*::Tn mutant cells were recovered when desiccated together (Fig. S7E). These findings raise a conundrum: growth to stationary phase is required for desiccation survival, yet the regulator most identified with stationary-phase growth (*rpoS*) is not required. We hypothesize that the stationary-phase phenotype(s) associated with desiccation survival is not controlled by RpoS. This may also reflect the somewhat diminished role that RpoS plays in P. aeruginosa stationary-phase survival relative to that in E. coli. Although P. aeruginosa RpoS regulates 772 genes in stationary phase, a deletion mutant is capable of withstanding some environmental stresses (49).

The current study sheds light on several genes and pathways required for P. aeruginosa tolerance to desiccation stress and/or recovery when the environment is rehydrated. When assessing the desiccation tolerance of P. aeruginosa relative to that of other organisms, the experimental conditions appear to be critical. While some studies find the desiccation tolerance of P. aeruginosa and that of E. coli to be comparable (16), a recent study found that E. coli was more resilient than P. aeruginosa (50). Both organisms, however, are less tolerant than A. baumannii, with strains that are capable of surviving desiccation periods up to 20 days (51, 52). Recent studies have begun to shed light on A. baumannii mechanisms of desiccation tolerance. A proteomics approach identified elevated protein levels for a number of cellular functions, including cell envelope integrity, protein stability and folding, oxidative stress resistance, and other stress responses (53). Some of those stress responses are likely controlled by the BfmR two-component response regulator (51). Though originally identified as a regulator of biofilm formation, a *bfmR* mutant is also desiccation intolerant. The authors propose that BfmR promotes desiccation tolerance through regulation of several stress response pathways. Another regulator critical for A. baumannii desiccation tolerance is the posttranscriptional regulator CsrA (50). Critical functions controlled by CsrA include catalase, a universal stress response protein, and two proteins of unknown function. Homologs of the latter proteins are absent from the P. aeruginosa genome (50). Whether those proteins partially account for the marked difference in desiccation tolerance between P. aeruginosa and A. baumannii and the mechanism by which they promote tolerance would be interesting future questions.

## MATERIALS AND METHODS

### Bacterial strains, plasmids, and primers.

The bacterial strains, plasmids, and primers used in this study are provided in Supplement A. The tetracycline resistance gene with its native promoter was PCR amplified from pEXG2Tc (54) using primers 292991923 and 292991924. The PCR product was introduced into pJN105 (55) digested with BspHI and NcoI, which removes the gentamicin marker, by Gibson cloning. The resulting plasmid was designated pJN105-Tc. The *surA* and *pdxA* expression vectors were constructed by Gibson cloning of PCR products generated using the indicated primers into pJN105-Tc digested with XbaI and SacI.

### Desiccation survival assays.

P. aeruginosa strain PA14 was grown overnight at 37°C with 200 rpm shaking in liquid LB for ~15 h. Cells were pelleted, washed, and resuspended in PBS to a final concentration of 10^8^ cells/mL. Cells (2 mL) were spotted onto the surface of 150 by 15 mm plastic petri dishes or 140 by 4 mm stainless-steel discs. The surfaces were desiccated in a biological hood for 3 h and then incubated at room temperature (RT) for 1, 3, or 7 days. The desiccated bacteria were rehydrated by adding 20 mL PBS at room temperature (RT) for 1 h. The cells were then removed from the surfaces using a cell scraper, serially diluted, plated on LB agar, and incubated for 24 h.

### Transposon mutant library preparation in Pseudomonas aeruginosa.

The transposon insertion mutant library was prepared as described previously (24, 56). Donor E. coli SM10 λpir harboring pBT20 plasmid (57) was inoculated in LB broth supplemented with ampicillin (100 μg/mL) and recipient P. aeruginosa strain PA14 inoculated in LB broth. Cells were cultured overnight at 37°C for ~15 h. Conjugations were initiated by adding 9 mL of the donor strain to 4.5 mL of the recipient, centrifuging at 5,000 × *g* for 10 min, and resuspending in 30 mL of 10 mM MgSO_4_. The mix was centrifuged, pelleted, and resuspended in PBS. The cell suspension was concentrated on four 0.45-μm nitrocellulose filters. The filters were placed on LB agar plates for 2.5 h at 37°C. Next, filters were placed into 35 mL MgSO_4_ and vortexed. The cell suspension was transferred to a new tube, pelleted, and resuspended in 5 mL MgSO_4_. One hundred microliters of the cells was plated on *Pseudomonas* isolation agar containing gentamicin (90 μg/mL), 50 plates, and incubated at 37°C for 18 h. Cells were collected in 15% glycerol LB broth, and aliquots were stored at −80°C. This biparental conjugation typically yielded ~7.5 × 10^7^ CFU.

### Tn-seq desiccation selection on plastic and stainless steel.

Aliquots of the PA14 Tn-seq library (2 mL in PBS at 3 × 10^8^ cells/mL) were spotted on the surface of either plastic or stainless steel in duplicate and desiccated as described above. After 1, 3, or 7 days, the viable cells were recovered in PBS as described above and an equal volume of 2× LB broth was added. Cells were outgrown at 37°C with 200 rpm shaking until the culture OD_600_ reached 0.5. The cells were pelleted for genomic DNA extractions. For the inputs/controls, 6 × 10^8^ cells were directly grown in LB broth without desiccation to an OD_600_ of 0.5.

### Tn-seq desiccation selection on plastic in the absence and presence of nutrients.

Aliquots of the PA14 Tn-seq library (10^9^ cells) were suspended in either 2 mL PBS (d-PBS) or 2 mL LB broth (d-LB), spotted on the surface of plastic dishes in duplicate, and desiccated as described above. After 1 or 3 days, viable cells were recovered in PBS or LB as described above and plated on VBM agar. After 24 h, the cells were collected and pelleted for genomic extractions.

### Tn-seq sequencing library preparation.

Libraries were prepared for sequencing as described previously with some modifications as outlined in Supplement B (24, 56). Genomic DNA was extracted using the DNeasy blood and tissue kit (Qiagen). Genomic DNA extracts were digested with KflI to eliminate sequence reads from integers and subsequently purified using a DNA clean and concentrator-5 kit (Zymo Research). A total of 100 ng of the genomic was linearly amplified using a dual priming oligonucleotide (268811662) and Q5 hot start high-fidelity DNA polymerase (New England Biolabs). Fifty PCR cycles were conducted with a 10-s extension in 50-μL reaction mixtures. Linear PCR products were purified as described above, c-tailed on the 3′ end, and purified. The linear c-tailed products were exponentially amplified using Q5 polymerase and the P5-Himar1-Bx (26881166x) and P716G p (273791651) primers (Table S5) for 22 to 25 PCR cycles in 25-μL reactions. The amplified libraries were fractionated on an agarose gel and stained with GelRed, and gel slices corresponding to 300 to 500 bp size were excised for gel purification. The libraries were sequenced on Hiseq X 150 PE.

### Data analyses.

A custom python script was used to demultiplex the libraries by searching for the 6-nucleotide barcodes upstream of the transposon sequence, with no mismatches permitted. The genomic junctions were extracted by removing the transposon inverted repeats and c-tails using TPP (25) with some modifications of the python script. Genomic junction sequence reads were mapped to Pseudomonas aeruginosa UCBPP-PA14 CP000438.1 using BWA 0.7.17-r1188 (58). The Prot_table annotation file was made from UCBPP-PA14 CP000438.1. Sequence reads, insertions, and gene fitness were obtained utilizing TRANSIT, resampling, and HMM (25).

### Competition assays.

Unmarked P. aeruginosa strain PA14 WT and PA14::Tn mutants (27) carrying gentamicin marker were grown overnight. The WT and indicated mutants were mixed 1:1 (10^8^ cells each) and desiccated as mentioned above. Desiccation tolerant cells were plated on LB agar and LB agar containing gentamicin. The competitive index was calculated as follows: CI = (number of recovered mutants from output/recovered WT from output)/(recovered mutants from input/recovered WT from input).

### Data availability.

Tn-seq sequencing bam files are available on NCBI Sequence Read Archive under BioProject number PRJNA783344. Data sets and Supplements A-B are available on Figshare: https://figshare.com/projects/Supplemental_information_and_data_sets/137101.
