# Supplementary figures and images for: Genome-Wide Identification of Pseudomonas aeruginosa Genes Important for Desiccation Tolerance on Inanimate Surfaces
Source: mSystems. 2022 Apr 26;7(3):e00114-22. doi: 10.1128/msystems.00114-22 (PMC9239045; doi:10.1128/msystems.00114-22)

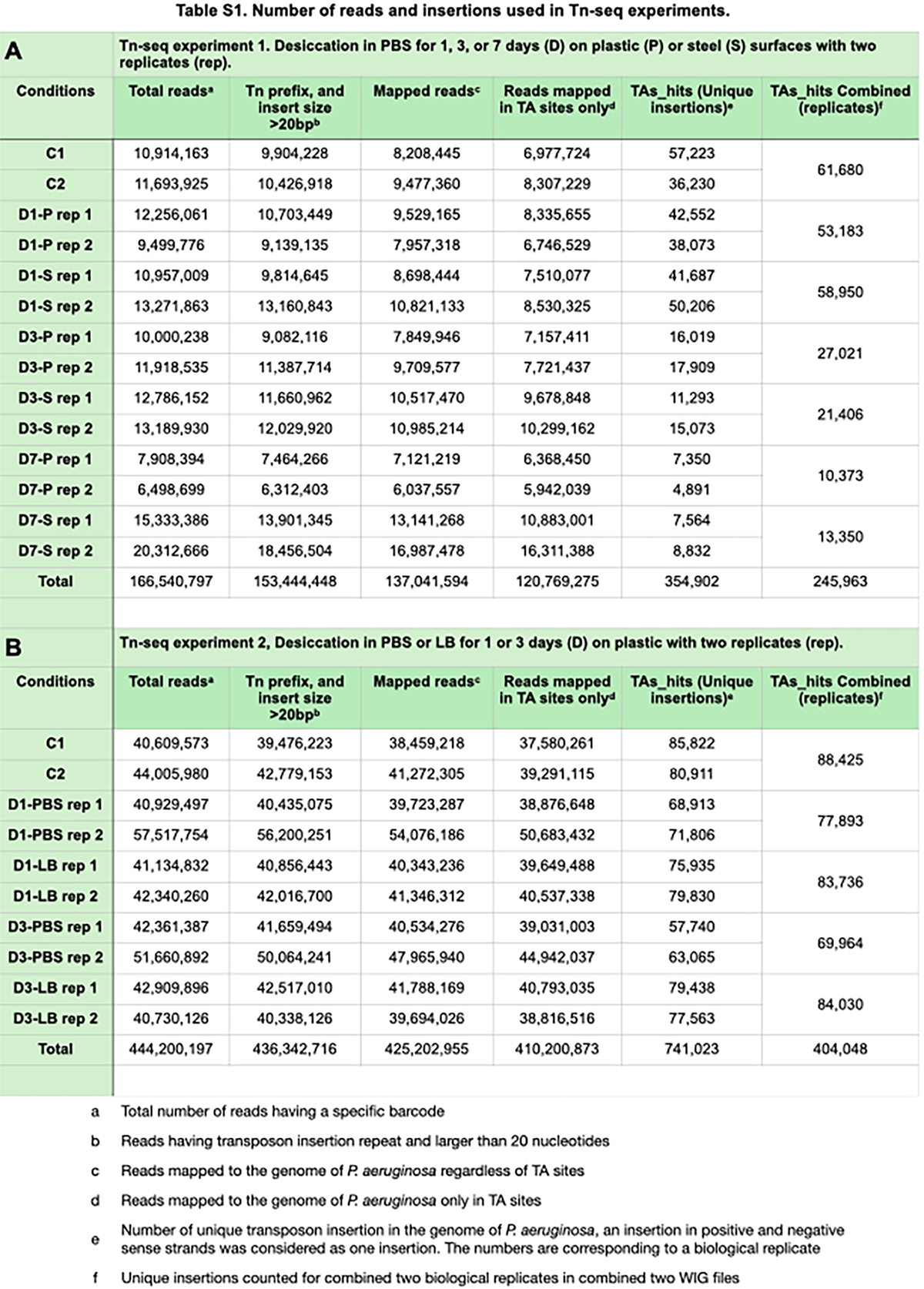

Supplement: TABLE S1 [file msystems.00114-22-s0008.tif]

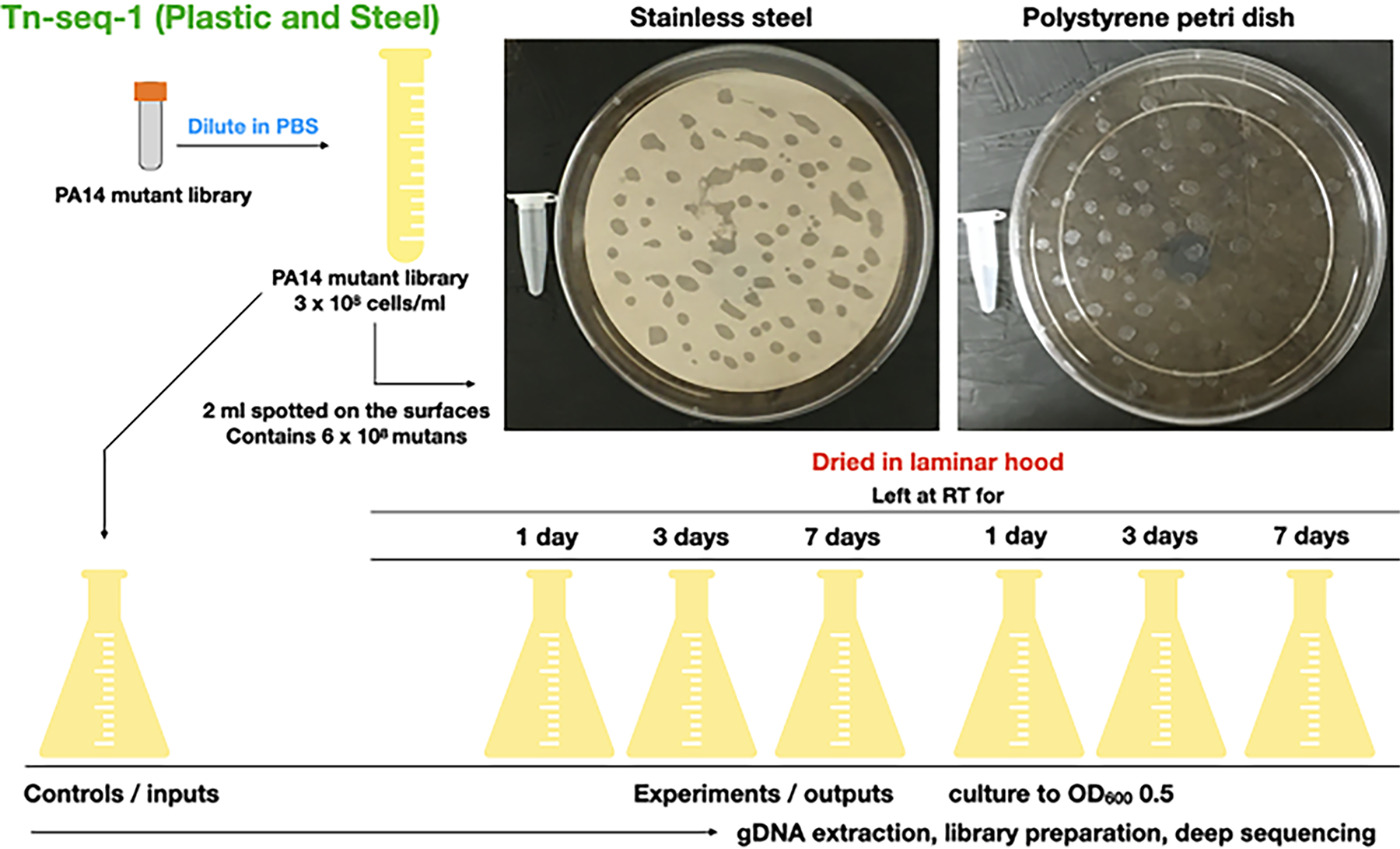

Supplement: FIG S1 [file msystems.00114-22-s0001.tif]

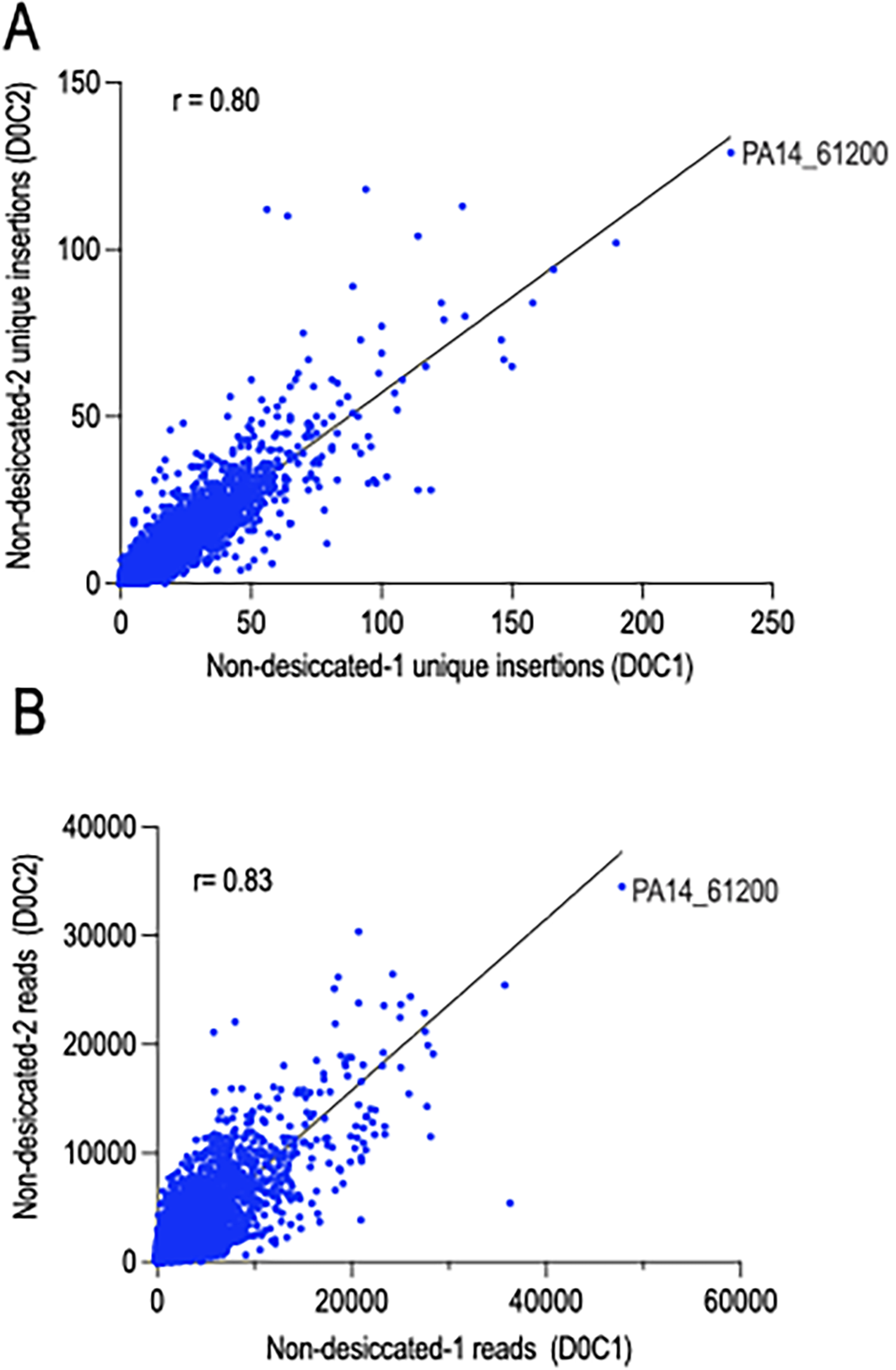

Supplement: FIG S2 [file msystems.00114-22-s0002.tif]

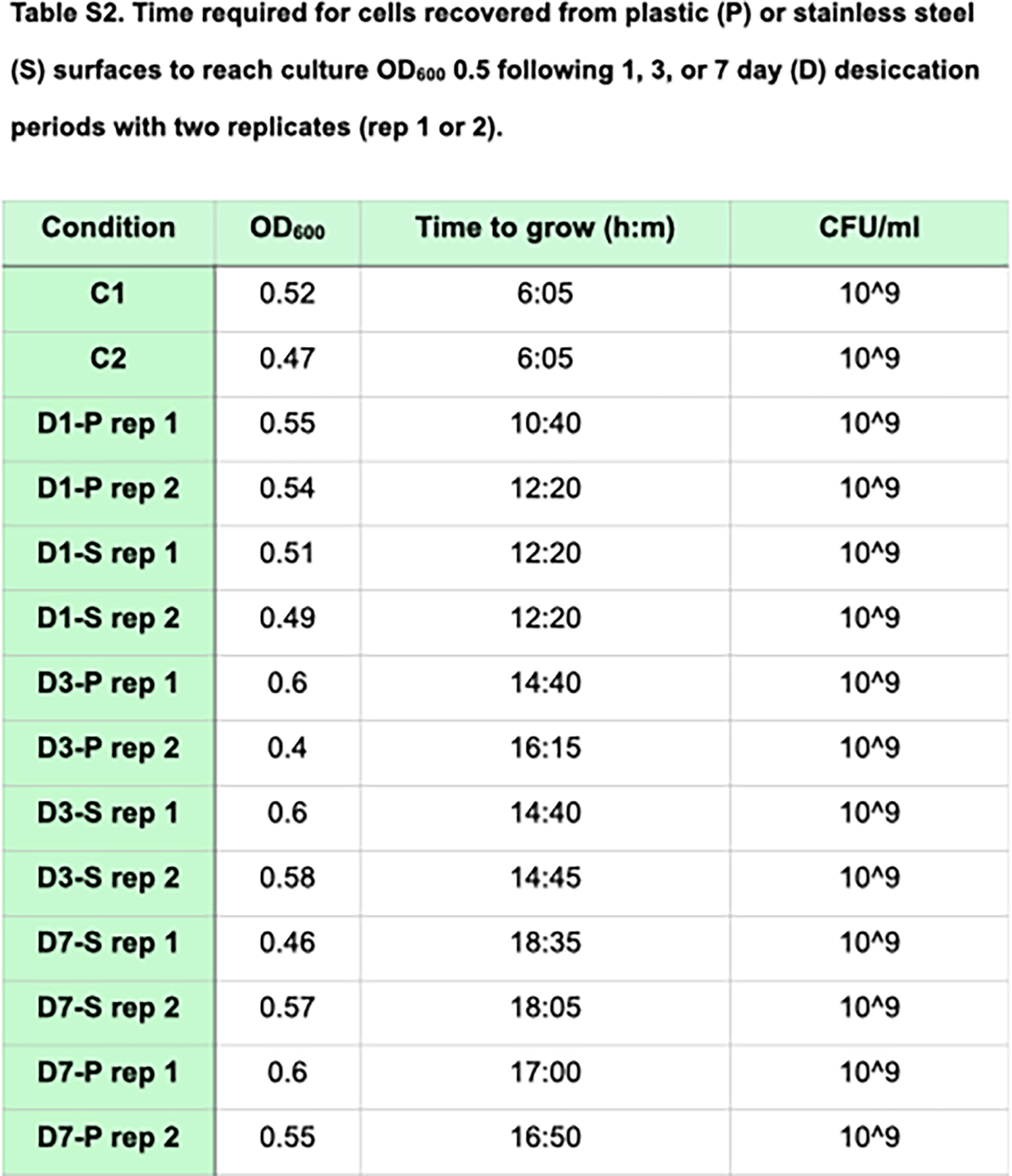

Supplement: TABLE S2 [file msystems.00114-22-s0009.tif]

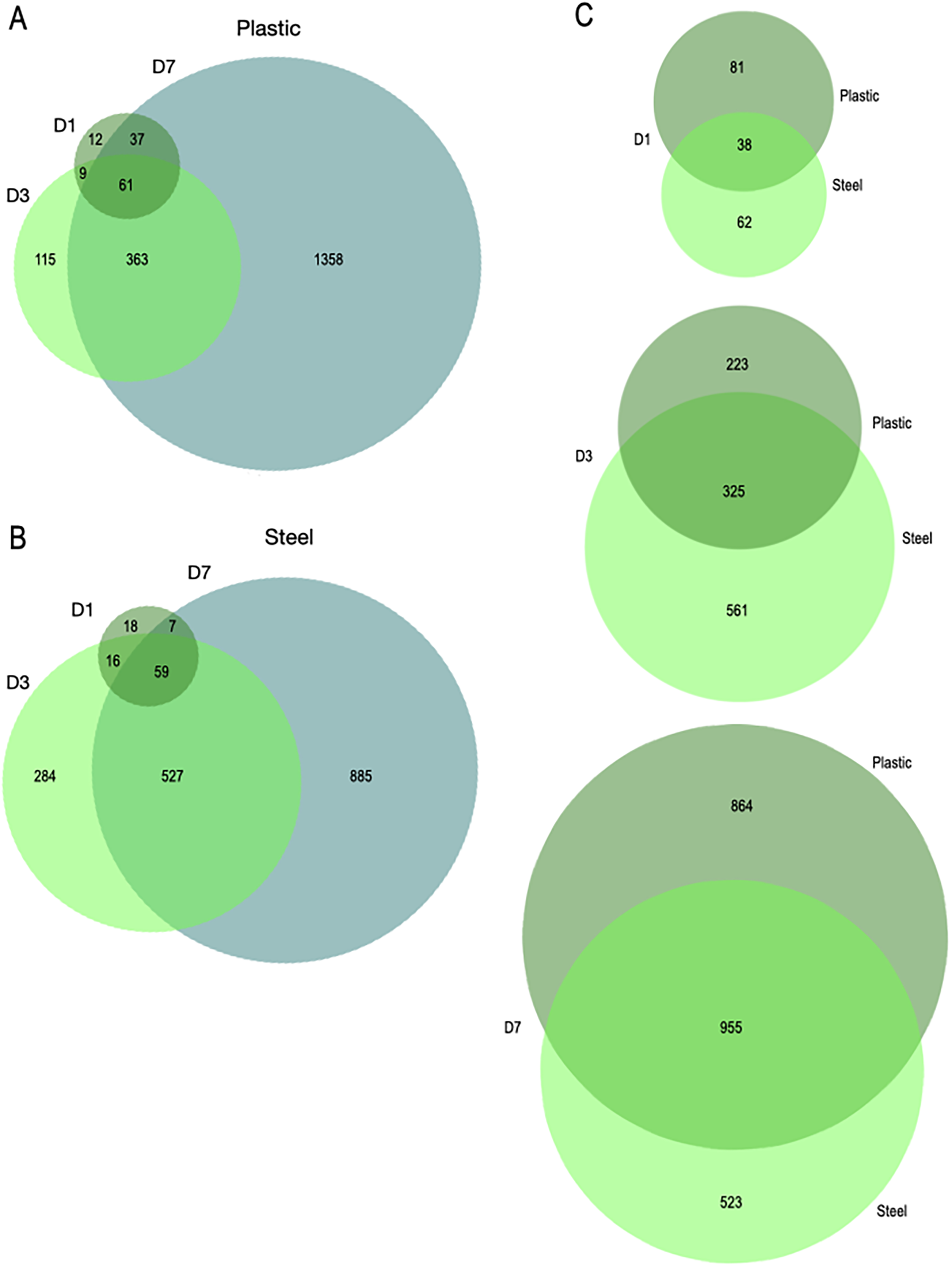

Supplement: FIG S3 [file msystems.00114-22-s0003.tif]

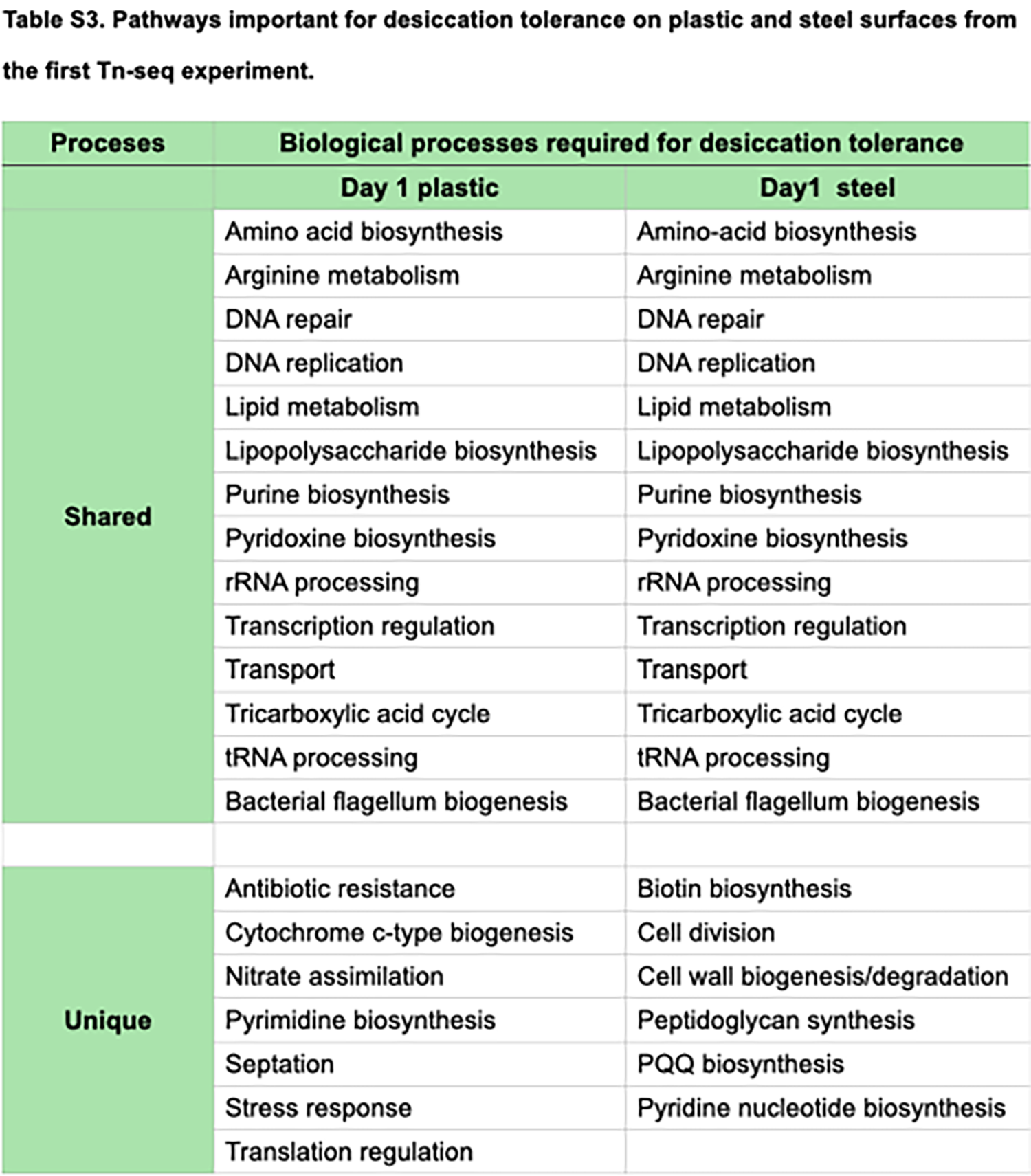

Supplement: TABLE S3 [file msystems.00114-22-s0010.tif]

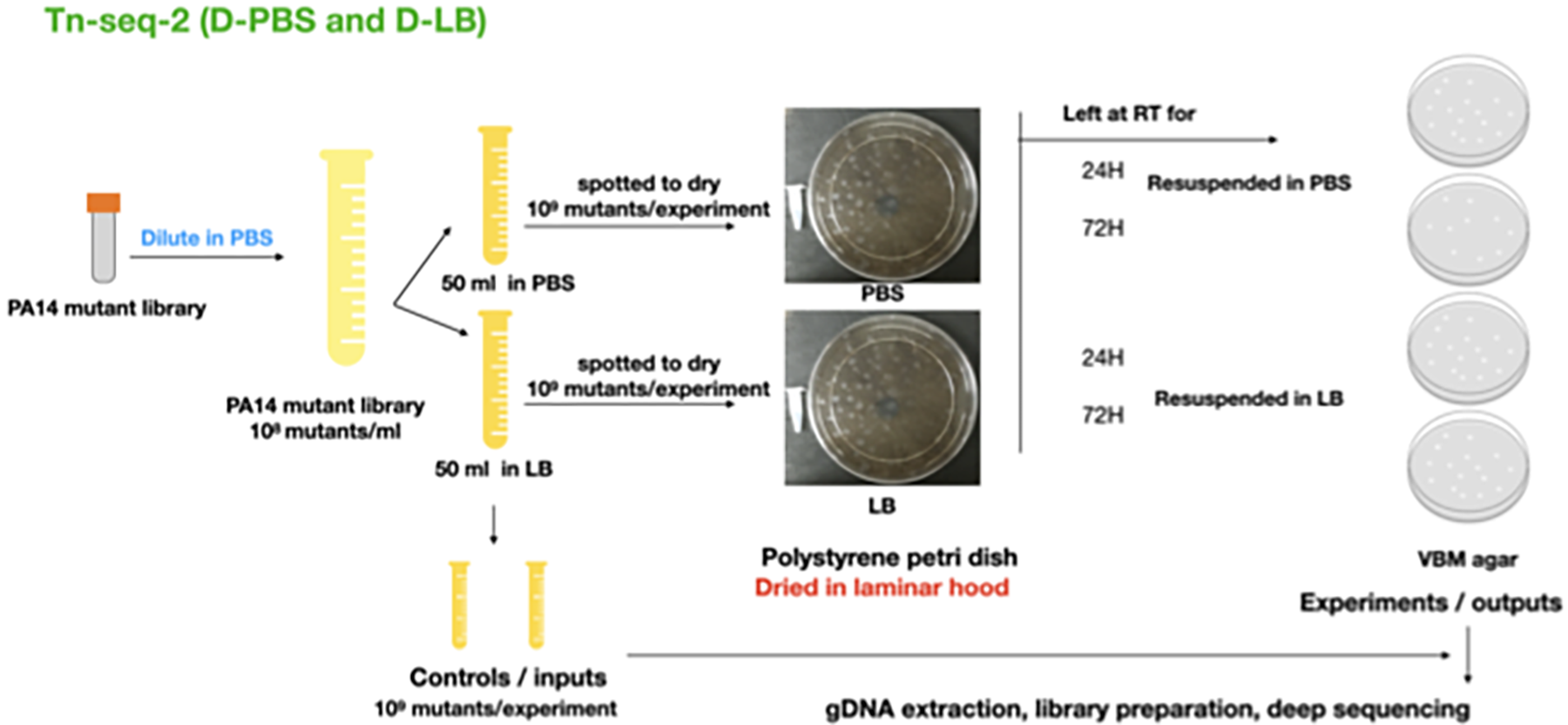

Supplement: FIG S4 [file msystems.00114-22-s0004.tif]

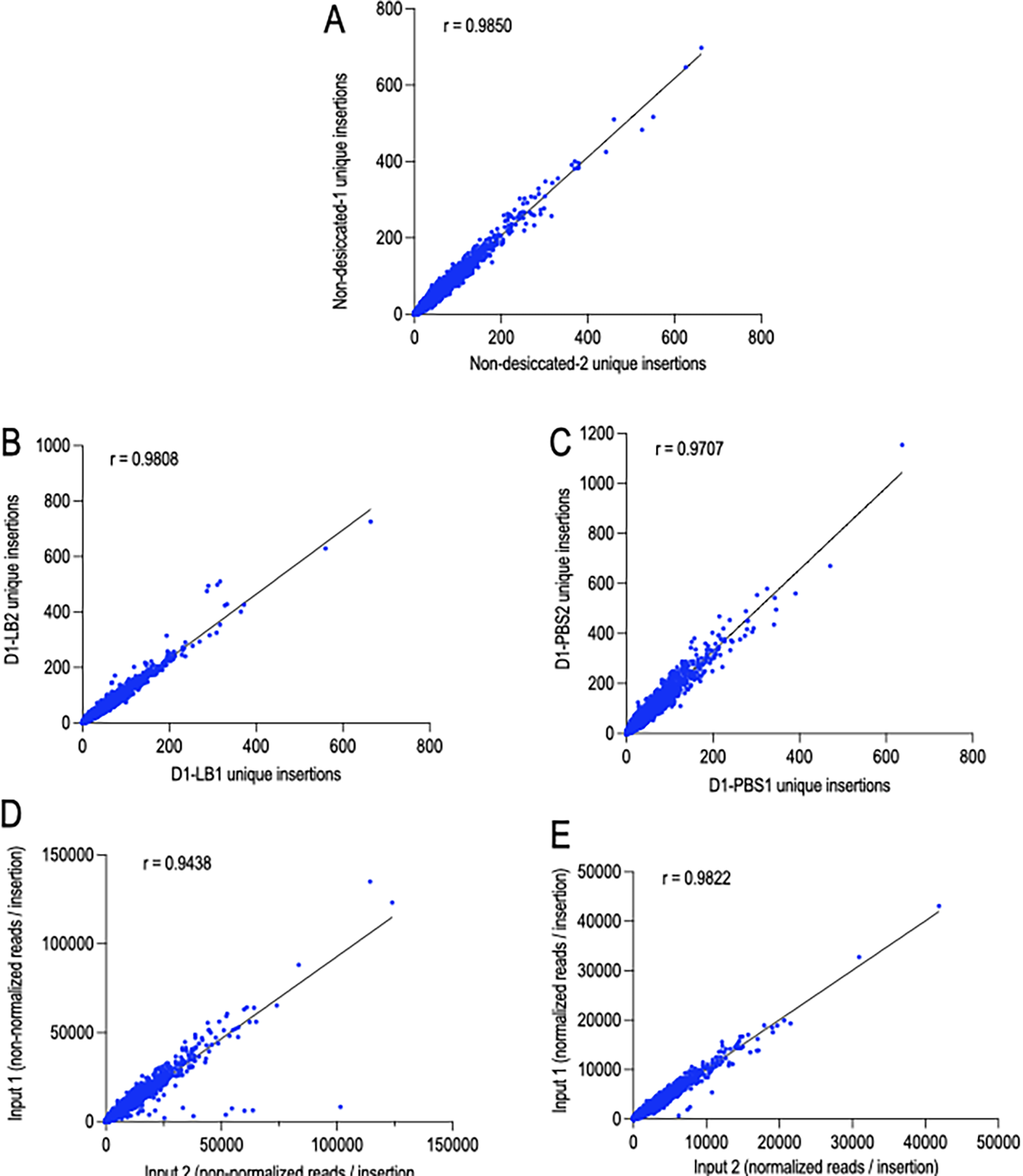

Supplement: FIG S5 [file msystems.00114-22-s0005.tif]

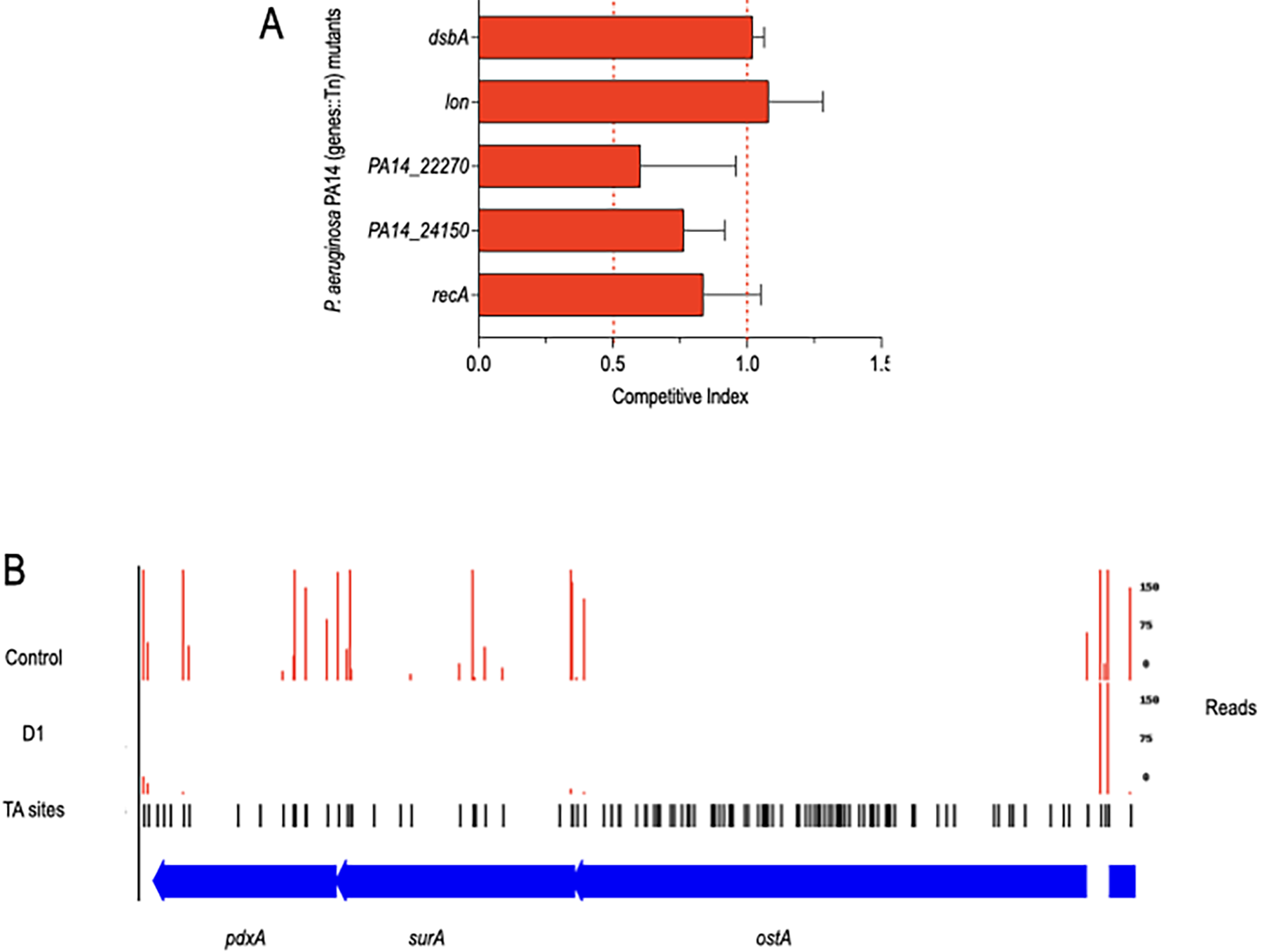

Supplement: FIG S6 [file msystems.00114-22-s0006.tif]

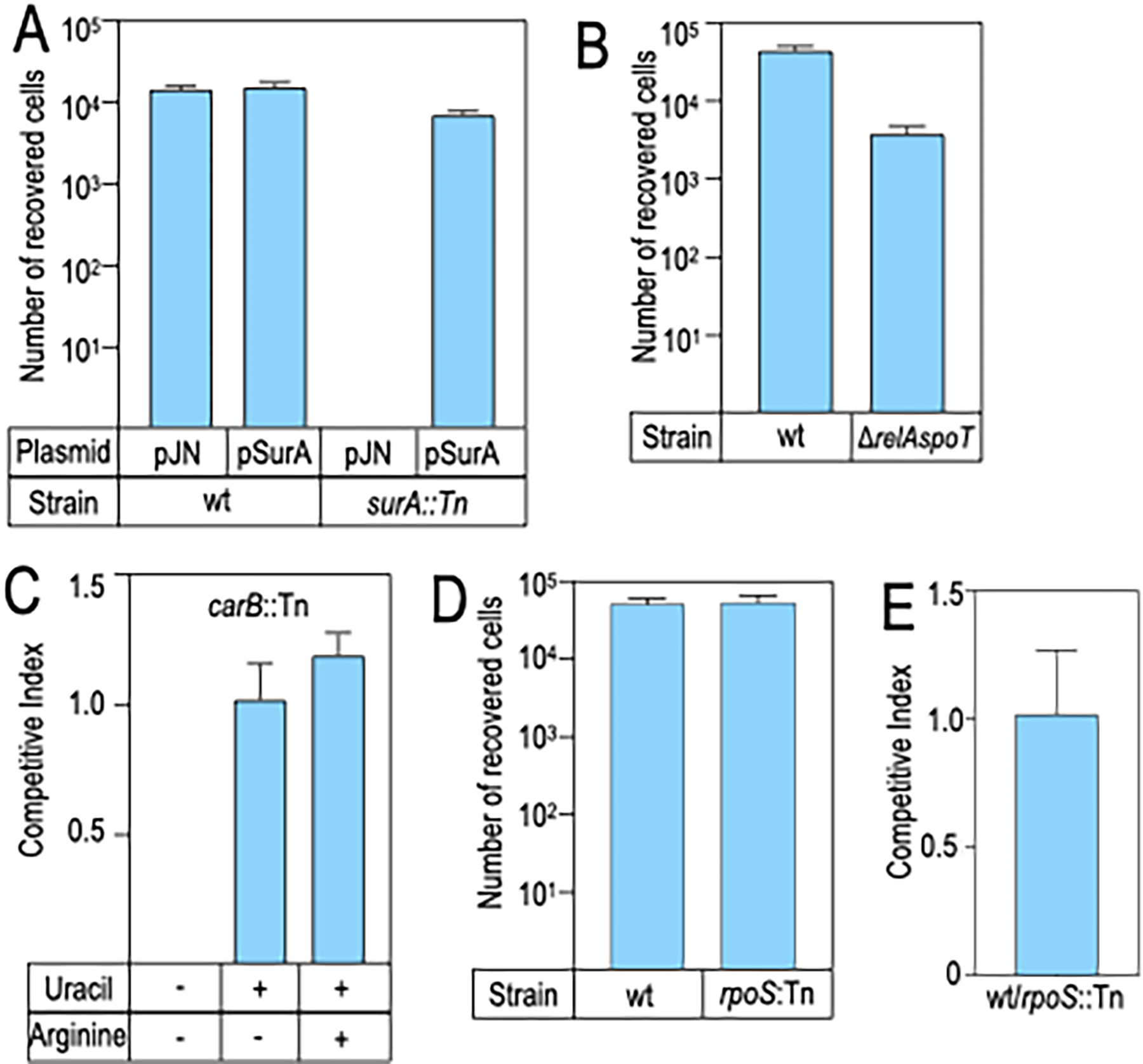

Supplement: FIG S7 [file msystems.00114-22-s0007.tif]
